# Trends of alcohol-attributable deaths in Lithuania 2001–2021: epidemiology and policy conclusions

**DOI:** 10.1186/s12889-024-18237-y

**Published:** 2024-03-12

**Authors:** Jürgen Rehm, Pol Rovira, Huan Jiang, Shannon Lange, Kevin D. Shield, Alexander Tran, Mindaugas Štelemėkas

**Affiliations:** 1 Institute for Mental Health Policy Research, Centre for Addiction and Mental Health, 33 Ursula Franklin Street, M5S 2S1 Toronto, ON Canada; 2 Campbell Family Mental Health Research Institute, Centre for Addiction and Mental Health, 250 College St, M5T 1R8 Toronto, ON Canada; 3 Dalla Lana School of Public Health, University of Toronto, 155 College Street, M5T 1P8 Toronto, ON Canada; 4 Institute of Medical Science, University of Toronto, Medical Sciences Building, 1 King’s College Circle, Room 2374, M5S 1A8 Toronto, ON Canada; 5 Department of Psychiatry, University of Toronto, 250 College Street, 8th floor, M5T 1R8 Toronto, ON Canada; 6 Centre for Interdisciplinary Addiction Research, Department of Psychiatry, University Medical Center Hamburg-Eppendorf, Martinistr. 52, 20246 Hamburg, Germany; 7 Program on Substance Abuse & WHO CC, Public Health Agency of Catalonia, 81-95 Roc Boronat St, 08005 Barcelona, Spain; 8 Health Research Institute, Faculty of Public Health, Lithuanian University of Health Sciences, Tilzes str. 18, 47181 Kaunas, Lithuania; 9 Department of Preventive Medicine, Faculty of Public Health, Lithuanian University of Health Sciences, Tilzes str. 18, 47181 Kaunas, Lithuania

**Keywords:** Alcohol, Attributable deaths, Fully attributable, Partially attributable, Alcohol-attributable deaths, Gender, Trends, Alcohol control policies, Lithuania

## Abstract

**Background:**

Lithuania, a Baltic country in the European Union, can be characterized by high alcohol consumption and attributable burden. The aim of this contribution is to estimate the mortality burden due to alcohol use for the past two decades based on different relative risk functions, identify trends, and analyse the associations of alcohol-attributable burden with alcohol control policies and life expectancy.

**Methods:**

The standard methodology used by the World Health Organization for estimating alcohol-attributable mortality was employed to generate mortality rates for alcohol-attributable mortality, standardized for Lithuania’s 2021 population distribution. Joinpoint analysis, T-tests, correlations, and regression analyses including meta-regressions were used to describe trends and associations.

**Results:**

Age-standardized alcohol-attributable mortality was high in Lithuania during the two decades between 2001 and 2021, irrespective of which relative risks were used for the estimates. Overall, there was a downward trend, mainly in males, which was associated with four years of intensive implementation of alcohol control policies in 2008, 2009, 2017, and 2018. For the remaining years, the rates of alcohol-attributable mortality were stagnant. Among males, the correlations between alcohol-attributable mortality and life expectancy were 0.90 and 0.76 for Russian and global relative risks respectively, and regression analyses indicated a significant association between changes in alcohol-attributable mortality and life expectancy, after controlling for gross domestic product.

**Conclusions:**

Male mortality and life expectancy in Lithuania were closely linked to alcohol-attributable mortality and markedly associated with strong alcohol control policies. Further implementation of such policies is predicted to lead to further improvements in life expectancy.

**Supplementary Information:**

The online version contains supplementary material available at 10.1186/s12889-024-18237-y.

## Background

Lithuania is the largest of the Baltic countries in the northeast part of the European Union (EU), and had 2.87 million inhabitants in 2023 [1]. Its alcohol *per capita* consumption (APC) among the population 15 years old and older has been among the highest globally in recent decades [2–4]; consequently, alcohol-attributable morbidity and mortality has been substantial in recent comparative risk assessments (CRA; [5, 6]). However, the most recently published global CRAs for alcohol use only cover the years 2016 [5] and 2019 (Global Burden of Disease Study: [6]). In the current investigation, we will provide alcohol-attributable mortality rates in Lithuania from 2001 up until 2021. Moreover, we will use data from Lithuanian cause-of-death certificates, which include all fully alcohol-attributable causes of death—including those which are too infrequent to be estimated in global databases—underlying the CRAs listed above (such as alcoholic polyneuropathy).

To determine alcohol-attributable mortality, we will use two different sets of risk relations. First, we will use the global relative risks (RRs) of the World Health Organization (WHO), which are used for the overwhelming majority of countries. These RRs have been derived from cause-specific meta-analyses, which are regularly updated (most recently for the upcoming report of Social Development Goal 3.5; for details, see Additional File 1). Second, we will use the so-called ‘Russian-specific’ RRs [7], which reflect a pattern of heavy-drinking occasions without daily drinking [8, 9]; these RRs have been customarily used for ex-Soviet Union countries in CRAs by the WHO [5]. The latter have been shown to be a slightly better fit for Lithuania than the global RRs when compared to direct estimates [10].

We will discuss whether the two sets of RRs yield different conclusions with respect to alcohol-attributable burden, and with respect to the impact of alcohol control policies (see also [11–13]). Given the high levels of alcohol consumption in Lithuania, we hypothesize: (a) a high correlation between alcohol-attributable mortality and life expectancy, and (b) marked associations between alcohol control policies and alcohol-attributable mortality and life expectancy.

The first hypothesis is based on prior high correlations between indicators for alcohol consumption and life expectancy in Eastern Europe (e.g., [14, 15]), based on the seemingly high association between alcohol use and cardiovascular disease (CVD) mortality in this region (e.g., [16]). The second hypothesis follows from the overall high effectiveness of the “best buys” policies not only in Lithuania, but in the region as a whole [10, 11].

### Methods

In general, we followed the WHO’s CRA methodology [17] to estimate mortality by alcohol-attributable causes of death by sex and age. There are two kinds of alcohol-attributable mortality—causes of death fully attributable to alcohol, such as alcohol use disorders, and alcoholic poisoning (for a full list, see [18]), and causes of death partially attributable to alcohol, such as stroke or breast cancer [18]. The former were taken directly from the death registries, the latter were calculated using the attributable-fraction methodology ( [19], see below). For causes of death partially attributable to alcohol, risk relations were taken from the WHO CRA (see [5]; and Additional File 1), either based on meta-analyses (global) or based on a large Russian retrospective case–control study ( [20]; for details on deriving the relative risk for Russia, see [5]).

### Data sources

#### General outline

Causes of death data were obtained by sex, age, and year from the Lithuanian Institute of Hygiene [21]. The results of the European Health Interview Survey of 2019 were retrieved from Statistics Lithuania [22]. Population by year was taken from Statistics Lithuania [23]. Gross domestic product (GDP) *per capita* was taken from the World Bank [24], and life expectancy at birth was taken from Statistics Lithuania [25] and from the World Bank [26].

#### Determining alcohol-attributable fractions

For death categories which are causally related to alcohol, the percentage attributable to alcohol was obtained by applying the alcohol-attributable methodology ( [19]; see Formula 1). The integral for current drinkers only considered consumption levels 150 g/day and below to avoid obtaining implausible values [27]. Any higher value was set to 150 g/day.

[**Formula 1**]

$$ AAF= \frac{{P}_{abs}{RR}_{abs}+ {P}_{form}{RR}_{form}+ {\int }_{0}^{150}{P}_{CD}\left(x\right){RR}_{CD}\left(x\right)dx-1 }{{P}_{abs}{RR}_{abs}+ {P}_{form}{RR}_{form}+ {\int }_{0}^{150}{P}_{CD}\left(x\right){RR}_{CD}\left(x\right)dx}$$

Where “x” is the average daily alcohol consumed in grams pure alcohol, “abs” is the abbreviation for abstainers, “form” is for former drinkers, and “CD” is for current drinkers. P_i_ represents the prevalence of each drinking group and RR_i_ represents its disease-specific RR. The prevalence of current drinkers (P_CD_(x)) is assumed to follow a gamma distribution with a mean value equal to the APC of the population [19, 28].

Since most assessment via self-reports, such as those collected via the use of surveys, markedly underestimate actual consumption [29, 30], survey data were triangulated with adult APC data as mainly derived from sales to determine alcohol exposure in grams (for justification, see [19, 28, 31]). Triangulation was done as follows: the overall level of consumption in a country was determined by the adult APC [32]. For our calculations, only 80% of the total adult APC were used to account for alcohol spilled and potential biases [31], as suggested by the Technical Advisory Group for Alcohol and Drug Epidemiology of the WHO, thus avoiding an overestimate. Survey estimates were taken from the most recent WHO comparative risk assessment for the years 2000–2020, based on all surveys collected from Lithuania [33]. The methodology to obtain these exposure estimates is described in Manthey and colleagues [34]. The values for the parameters of Formula 1 obtained by using this methodology for each year can be found in Additional File 2. For most causes of death, no lag time was assumed, but cancers were modelled with a 10-year lag time since exposure [35].

Uncertainty around all point estimates was estimated using a Monte Carlo simulation [36] with 1,000 repetitions. This Monte Carlo simulation randomly took values for all parameters in Formula 1 with the probability of this selection based on the density of the random error distribution around the point estimate (i.e., highest probability for point estimate, for values different from the point estimate based on the density distribution around the point estimate).

#### Describing trends of alcohol-attributable mortality

To ensure comparable data over time, we age-standardized the alcohol-attributable mortality rates to the Lithuanian population distribution of 2021. To determine inflection points for trends, we used joinpoint analysis [37]. This statistical analysis used trend data and fits the simplest model that the data allows for, starting with the minimum number of inflection points (for example, no inflection point is associated with a straight line) and tests whether more such points are statistically significant and should be added to the model up to the maximum number the data allow for. For theoretical reasons (two 2-year intervention periods with alcohol control policy), we tested up to 5 joinpoints.

#### Establishing associations between alcohol-attributable mortality and alcohol control policies and life expectancy

We selected the years 2008 and 2009, and the years 2017 and 2018, as years in which significant alcohol control policies were implemented, since it was in these four years that the WHO best-buy policies were enacted—these policies had been hypothesized to have immediate impact on mortality (taxation policies that resulted in decreased affordability and an availability reduction of at least 20%; for a selection of interventions, see [38, 39]; for impact on mortality, see [40]). The association with the various alcohol control policies was established by measuring the reduction in alcohol-attributable mortality from the year prior to the policy’s implementation to its enactment date and then comparing the difference to the average change in other years. This comparison was done with and without taking into account the variance of the point estimates for alcohol-attributable mortality, the former via meta-regression [41].

Finally, we measured associations of the alcohol-attributable mortality rate with life expectancy at birth, by sex, using Pearson correlation coefficients. Additionally, to avoid spurious correlations, regression analyses were conducted predicting differenced sex-specific life expectancy by differenced alcohol-attributable mortality rates, controlled for GDP *per capita*.

All statistical analyses were conducted using R version 4.2.0 [42] and were performed independently by sex, age, and disease type.

## Results

### Levels and trends in alcohol-attributable mortality

**Fig. 1:**
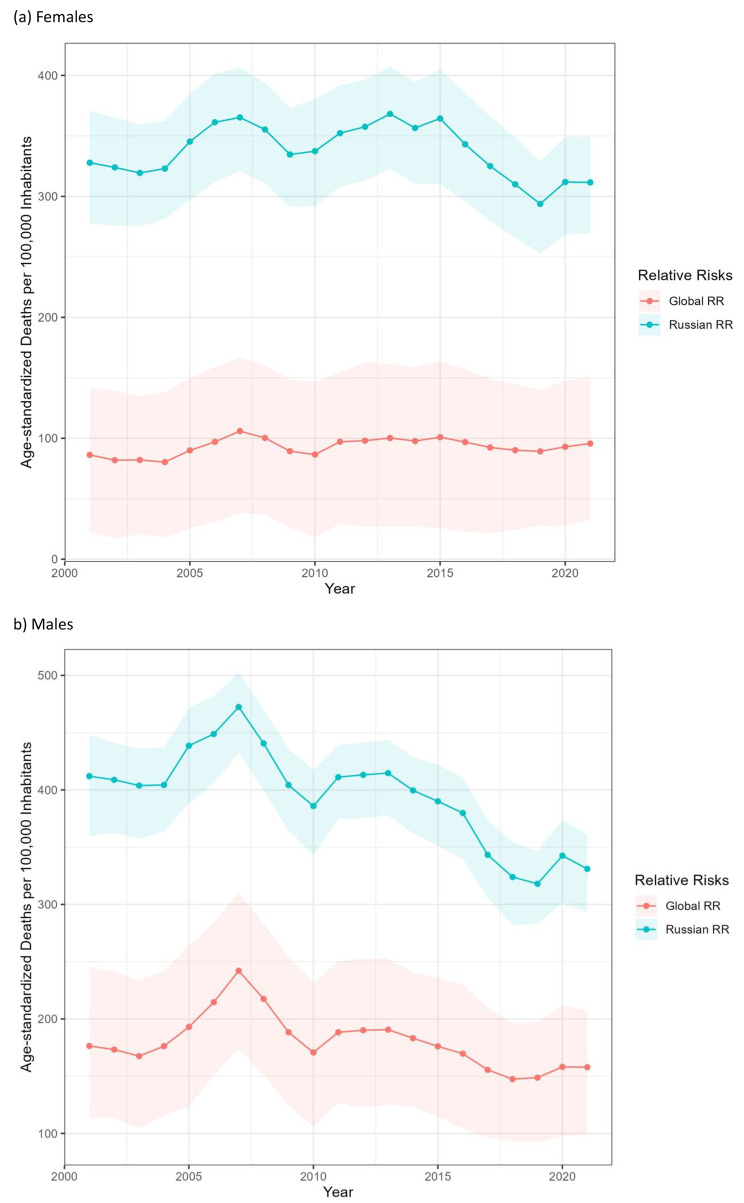
Age-adjusted alcohol-attributable mortality rates per 100,000 adults in Lithuania

Figure 1a and b give the trends of age-adjusted alcohol-attributable mortality rates per 100,000 population over 15 years of age from 2001 to 2021.

Overall, Lithuanian alcohol-attributable mortality is among the highest in the EU [2, 17], irrespective of the RRs used (see also the alcohol-attributable fractions in Additional File 3). As expected, males have higher alcohol-attributable mortality compared to females, and the Russian RRs lead to higher mortality estimates compared to the global RRs. In addition, Fig. 1 shows that males have higher variability of estimates regardless of which RRs are used. For both sexes, alcohol-attributable mortality increased in the early years of the 21st century until 2007, which is one inflection point, after which alcohol-attributable mortality decreased. Moreover, after another upturn in the early years of the second decade, there seems to have been another inflection point in the mid-2010s, lasting until 2020 when the trends changed again in the first year of the COVID-19 pandemic. The formal joinpoint analyses confirm these trends from visual inspection (for details, please see Additional File 4). Finally, we see an overall downward trend in alcohol-attributable mortality in men, but not necessarily in women (males, Global RR, -1.90, 95%CI: -3.47, -0.33 age-standardized deaths per 100,000; females, Global RR, 0.90, 95%CI:-0.05, 0.93; males, Russian RR, -5.16, 95%CI: -7.27,-3.05; females, Russian RR, 0.44, 95%CI: -2.51, 0.72).

### Trends, relative risks used, and gender

Some of the gender specificities have been described above. Table 1 provides further detail on gender specificities, which also allow us to highlight some of the underlying assumptions for the RRs used. First, there are marked gender difference in all indicators examined except for the overall attributable fraction based on Russian RRs (see Table 1). The proportion of fully alcohol-attributable deaths in males was more than double than for females. The largest proportion here would be alcohol-attributable liver deaths and alcohol poisonings (74% of all 100% alcohol-attributable deaths for both sexes, 70.9% for males, 78.1% for females). This is in line with heavy-drinking occasions indicated in the last Lithuanian iteration of the European Health Interview Survey of 2019 [22], where males indicated a more than fourfold prevalence of heavy drinking at least monthly (see Table 1). However, these indicators do not seem to be in line with the overall alcohol-attributable fraction, i.e., the percentage of alcohol-attributable deaths out of all deaths based on Russian RRs, where females show higher proportions than males, and an overall very high proportion of alcohol-attributable deaths (21.2%). As alcohol-attributable CVD deaths are particularly related to frequent heavy-drinking occasions [43, 44], which are comparatively low in females (see Table 1), we conclude that the Russian RRs are likely to be an overestimate for Lithuanian females (see Discussion below).

**Table 1:** Indicators of alcohol-attributable mortality 2001–2021 by gender

	% fully AA deaths out of all deaths	% AA deaths out of all deaths– RUS RRs	% AA deaths out of all deaths– global RRs	% CVD deaths in AA deaths– RUS RRs	% CVD in AA deaths– global RRs	% at least monthly/weekly heavy-drinking occasions in 2019 among drinkers*
Females	0.9%	21.2%	5.9%	82.0%	57.7%	6.5%/0.4%
Males	2.6%	20.3%	9.5%	43.6%	2.6%	27.4%/5.1%

AA: alcohol-attributable; RUS: Russian; CVD: cardiovascular disease;

*European Health Interview Survey of 2019; data retrieved from Statistics Lithuania [22].

### Associations between alcohol-attributable mortality and major alcohol control policies, and between alcohol-attributable mortality and life expectancy

During the time periods of observation, there were two periods of two years each in which major alcohol control policies were enacted, including the WHO “best buys” (for a definition, see [45]; for operationalization in Lithuania, see [38, 39]). In these periods—2008/2009, and 2017/2018—all of the policies were implemented in the first quarter (January or March) of the respective year, so we would expect a strong immediate effect lasting for at least one year. We tested the potential associations of alcohol-attributable mortality rates with alcohol control policies by comparing percentage changes in these rates from each year to the following year. The above-specified years with major alcohol policies were all linked to a reduction in alcohol-attributable mortality (larger for males) and irrespective of the underlying RRs, whereas for all remaining years alcohol-attributable mortality increased on average. In other words, alcohol-attributable mortality seems to be impacted by alcohol control policies. This association was shown to be statistically significant using a group comparison t-test on the point estimates (see Additional File 6 for details). However, when taking into account the variance of the point estimates for alcohol-attributable mortality and giving more weight to those estimates with smaller variance by using meta-regression, the differences were no longer statistically significant.

As for the link of alcohol-attributable mortality to life expectancy, the correlation was both high and significant for men (Russian RRs: Pearson’s *r* = -0.90, 95% CI: -0.95, -0.76, Global RRs: Pearson’s *r* = -0.76, 95% CI: -0.90, -0.49), but not for women (Russian RRs: Pearson’s *r* = -0.30, 95% CI: -0.65, 0.14, Global RRs: Pearson’s *r* = 0.16, 95% CI: -0.29, 0.55). These associations for men remained significant after controlling for economic wealth using differenced series for both life expectancy and alcohol-attributable mortality (see Additional File 5). Also, for males, these differences series showed no autoregression.

## Discussion

Although there are some indications of an overall downward trend, alcohol-attributable mortality was high in Lithuania during the two decades from 2001 to 2021 (between 80 and 500 deaths per 100,000 population), irrespective of which RRs were used for the estimates. The overall downward trend can be attributed to four years of intensive implementation of alcohol control policies in 2008, 2009, 2017 and 2018; for the remaining years, the rates of alcohol-attributable mortality were stagnant. Among males, the correlations between alcohol-attributable mortality and life expectancy were − 0.90 and − 0.76 for Russian and global RRs respectively; and persisted in regression analyses using differenced series for life expectancy and alcohol-attributable mortality adjusted for GDP *per capita* as a potential confounder.

Before we discuss these results further, we need to acknowledge some limitations of the approach used. The methodology of CRAs is based on the attributable-fraction methodology, which has a number of crucial assumptions which may not hold in the given context [46]. Most importantly, as for all global CRAs (e.g., [5, 6]), we based our estimates on global or regional RRs, which may not be accurate enough for the Lithuanian context of the past two decades. In fact, in a recent comparison with direct estimates in Lithuania—based on reductions in population-level consumption due to alcohol control policies—showed that both types of estimates used (i.e., Russian and global RRs) deviated substantially and underestimated the alcohol-attributable mortality burden for men [10]. As well, the Russian RRs may have been based on hazardous drinking patterns of surrogate alcohol, which had been common in Russia at the time of the Zaridze study [47, 48], but much less so in the Baltic countries over the past 20 years [49]. Clearly, we need better and more country-specific RRs. The final limitation is the use of Pearson’s correlation coefficient to measure the association between two time-series, which could be subject to spurious correlation. To guard against this risk, we additionally conducted regression analyses with differenced data, and included GPD *per capita* as a potential confounder. The strong relationships between alcohol-attributable mortality and life expectancy for males persisted.

Key to the development of a country-specific estimate for Lithuania seems to be the relationship between alcohol use and cardiovascular mortality. Here, the different RRs used showed tremendous differences (see above). And the literature also points to differences in RRs for the level of alcohol use and CVD categories between Western and Eastern European countries [7, 50, 51]. For males, Lithuania seems to clearly align with Eastern European countries such as Russia [16], showing marked associations between alcohol use and CVD. This has also been corroborated using a different direct methodology approach examining changes after major policy implementation. Stumbrys and colleagues [13] showed that the typical increases in CVD mortality on Mondays, which had been found in Lithuania and other post-Soviet countries

[52–54], disappeared after alcohol sales were restricted on Sundays. Manthey and colleagues [55] showed that alcohol taxation increases were associated with reduced mortality inequalities, with a large proportion of these resulting from CVD. Finally, using the same taxation increase, Tran and colleagues showed that cardiovascular causes of death ( [56]; see also [10]) were the largest contributor to the changed all-cause mortality rate. While these analyses clearly show the impact of alcohol control policies on male CVD mortality in Lithuania, we would need more cohort data in representative populations on alcohol use and CVD, including populations with higher-risk drinking patterns than commonly found in cohort studies [57]. It may also be that drinking patterns which play a crucial role for CVD mortality (e.g., [44, 47]) have changed over the past two decades.

While we refrained from using causal terminology to describe our results which are based on correlations and ecological associations, we switched to such terminology in the final paragraph when we referred to well-controlled time-series analyses used to test *a priori*-specified hypotheses (for further reasoning, see [58]). We believe this switch is justified given the underlying methodology, and the well-developed overall evidence for the effectiveness of availability restrictions and taxation increases in lowering alcohol consumption [59–61].

To be conservative and consistent with prior analyses (e.g., [40, 62, 63]), we restricted the impact of policies in this paper to last for one year only. While the impact of alcohol control policies will usually last longer, depending on changes in inflation, affordability, and adaptation to availability restrictions, it is hard to determine the magnitude of the lagged effects. Alcohol policy-makers should implement additional elements which guard against eroding effects (e.g. by linking taxation rates to inflation or affordability measures [59, 64]).

In the absence of Lithuania-specific RRs, and based on the current evidence, we argue that Russian RRs better describe the CVD burden in males, due to their high prevalence of episodic heavy drinking— especially important for ischemic disease [44, 54] which has been linked to an overall irregular drinking pattern with many abstinent days but a high overall level of consumption [13, 65]. We further base our argument on the direct results of earlier studies which have shown an impact from the implementation of major alcohol control policies (see examples in the last paragraph). This would also be consistent with the high proportion of 100% (i.e., fully) alcohol-attributable disease categories among males (see Table 1). On the other hand, the current drinking pattern and results from the direct studies [10] indicate that for females the global RRs should be used until better country-specific evidence from the above-described cohort studies can be obtained. Given the similarity of effects of policies on consumption and alcohol-attributable harm in the two other Baltic countries [11], the same solution may hold for Estonia and Lativia as well.

Life expectancy in Lithuania is comparatively low compared to the rest of the EU, but for males this is true even when compared to neighbouring countries such as Estonia [26, 66]. In 2021, Lithuanian males had a life expectancy of almost 8 years lower than the EU average, with a smaller gap for females of four years. The gender gap in life expectancy in Lithuania is also one of the highest in the EU (see also [67]). Together with the results of the overall evaluation of alcohol control polices [11], the results presented indicate that one way to decrease this gap for males could be the further implementation of alcohol control policies. Given the high level of alcohol-attributable burden in males, and the high correlation with life expectancy, lowering alcohol-attributable burden would result in narrowing this gap. In particular, analyses of taxation increases indicated a sizable decrease in all-cause mortality in males [40, 56], thus increasing overall life expectancy.

## Conclusions

In conclusion, the alcohol-attributable mortality rate in Lithuania has been high over the past two decades, and this is reflective of the country’s high APC. In Lithuania, males consume a notably higher amount of alcohol compared to females, and, as such, the finding that male alcohol-attributable mortality rate was more closely linked to their life expectancy compared to females was to be expected. Taken together with the results of the evaluation of alcohol control policies [11], it is suggested that the implementation of alcohol control policies— including availability restrictions and taxation increases—is a good way to increase life expectancy among Lithuanian males in particular. Future studies on alcohol use and CVD are necessary, but, in the meantime, we suggest using Russian RRs for alcohol studies involving Lithuanian males, while global RRs should be used for Lithuanian females.

### Electronic supplementary material

Below is the link to the electronic supplementary material.

Supplementary Material 1

## Data Availability

The main underlying data (deaths by cause of death, sex, age and time of death) can be downloaded from the Lithuanian Institute of Hygiene (under the Ministry of Health): https://stat.hi.lt/default.aspx?report_id=204. All R codes used in the analyses can be obtained from the first author upon request.

## Declarations

The study protocols were approved by the Research Ethics Board of the Centre for Addiction and Mental Health, Toronto, Canada on April 17, 2020, and renewed every year (REB decision letter no. 050/2020).

## Abbreviations

APC: Adult alcohol *per capita* Consumption
CRA: Comparative Risk Assessment
GDP: Gross Domestic Product
RR: Relative Risk
WHO: World Health Organization
