# Supplementary material for: Trends of alcohol-attributable deaths in Lithuania 2001–2021: epidemiology and policy conclusions
Source: BMC Public Health. 2024 Mar 12;24:774. doi: 10.1186/s12889-024-18237-y (PMC10935848; doi:10.1186/s12889-024-18237-y)
Supplement: Supplementary file 1 — Supplementary Material 1 [file 12889_2024_18237_MOESM1_ESM.docx]

# Additional Files

Table of Contents

[Additional Files 1](#_Toc156989141)

[Additional File 1: Global and Russian Relative Risks for partially attributable disease categories 2](#_Toc156989142)

[Table AF1: Global Relative Risks for partially attributable disease categories 2](#_Toc156989143)

[Table AF2: Relative Risks for partially attributable disease categories for Russia and similar countries 9](#_Toc156989144)

[References 12](#_Toc156989145)

[Additional File 2: Parameters for the alcohol-attributable fraction models 14](#_Toc156989146)

[Additional File 3: Overall alcohol-attributable fractions for Lithuania 2001-2021, based on different relative risks 16](#_Toc156989147)

[Figure AF1: Overall alcohol-attributable fractions by sex 16](#_Toc156989148)

[Additional file 4: Joinpoint analyses 18](#_Toc156989149)

[Joinpoint analysis (Male, Russian relative risk) 18](#_Toc156989150)

[Joinpoint analysis (Male, Global relative risk) 19](#_Toc156989151)

[Joinpoint analysis (Female, Global relative risk) 20](#_Toc156989152)

[Joinpoint analysis (Female, Russian relative risk) 21](#_Toc156989153)

[Additional file 5: Yearly changes in alcohol-attributable mortality, associations with life expectancy 22](#_Toc156989154)

[Yearly change in alcohol-attributable mortality (males, Global RR) 22](#_Toc156989155)

[Yearly change in alcohol-attributable mortality (females, Global RR) 22](#_Toc156989156)

[Yearly change in alcohol-attributable mortality (males, Russian RR) 22](#_Toc156989157)

[Yearly change in alcohol-attributable mortality (females, Russian RR) 22](#_Toc156989158)

[Difference in life expectancy predicted by GDP and difference in alcohol-attributable mortality (males Global RR) 23](#_Toc156989159)

[Difference in life expectancy predicted by GDP and difference in alcohol-attributable mortality (females Global RR) 23](#_Toc156989160)

[Difference in life expectancy predicted by GDP and difference in alcohol-attributable mortality (males, Russian RR) 23](#_Toc156989161)

[Difference in life expectancy predicted by GDP and difference in alcohol-attributable mortality (females, Russian RR) 24](#_Toc156989162)

[Additional file 6: Measuring the impact of alcohol control policies by RR type and sex 25](#_Toc156989163)

## Additional File 1: Global and Russian Relative Risks for partially attributable disease categories

### Table AF1: Global Relative Risks for partially attributable disease categories

| Disease | Sex / Age | Relative risk function (where x is the daily alcohol consumption in g) | Source |
| --- | --- | --- | --- |
| Tuberculosis (ICD-10 coding: A15-19, B90) | Both / All ages | RR_CD_ = exp(x∙β1)  β1 = 0.0179695 | Imtiaz et al., 2017 (1) |
| HIV/AIDS (ICD-10 coding: B20-24) | Males / All ages | if(x ≤ 61) RR_CD_ = 1  if(61 > x) RR_CD_ = β1  β1 = ln(1.54) | Rehm et al., 2017 (2) |
|  | Females / All ages | if(x ≤ 49) RR_CD_ = 1  if(49 > x) RR_CD_ = β1  β1 = ln(1.54) | Rehm et al., 2017 (2) |
| Lower respiratory infections (ICD-10 coding: J09-22, P23, U04) | Both / All ages | RR = exp(β1∙( (x + 0.0399999618530273) / 100) )  β1 = 0.4764038 | Samokhvalov et al., 2010 (3) |
| Lip and oral cavity cancer  (ICD-10 codes: C00-08) | Both / All ages | RR_CD_ = exp(x∙β1 + x^2^∙β2)  β1 = 0.02474  β2 = -0.00004 | Bagnardi et al., 2015 (4) |
| Other pharyngeal cancers  (ICD-10 codes: C09-10,  C12-14) | Both / All ages | RR_CD_ = exp(x∙β1 + x^2^∙β2)  β1 = 0.02474  β2 = -0.00004 | Bagnardi et al., 2015 (4) |
| Oesophagus cancer  (ICD-10 codes: C15) | Both / All ages | RR_CD_ = exp(x∙β1 + x^2^∙β2)  β1 = 0.0132063596418668  β2 = -4.14801974664481*10^-08^ | Bagnardi et al., 2015 (4) |
| Colon and rectum cancers  (ICD-10 codes: C18-21) | Both / All ages | RR_CD_ = exp(x∙β1)  β1 = 0.006765865 | Vieira et al., 2017 (5) |
| Liver cancer  (ICD-10 codes: C22) | Both / All ages | RR_CD_ = exp(x∙β1)  β1 = 0.003922071 | World Cancer Research Fund International, 2018 (6) |
| Larynx cancer  (ICD-10 codes: C32) | Both / All ages | RR_CD_ = exp(x∙β1 + x^2^∙β2)  β1 = 0.01462  β2 = -0.00002 | Bagnardi et al., 2015 (4) |
| Breast cancer  (ICD-10 codes: C50) | Females / All ages | RR_CD_ = exp(x∙β1)  β1 = 0.018232156 | Sun, 2020 (7) |
| Cervix uteri cancer  (ICD-10 codes: C53) | Females / All ages | if(x ≤ 49) RR_CD_ = 1  if(49 > x) RR_CD_ = β1  β1 = ln(1.54) | Rehm et al., 2017 (2) |
| Diabetes mellitus (ICD-10 coding: E10–E14 (minus E10.2–E10.29, E11.2–E11.29, E12.2, E13.2–E13.29, E14.2)) | Male / All ages | RR_CD_ = exp(x∙β1)  β1 = ln(1.18) | World Health Organization 2023 (8) |
|  | Females / All ages | RR_CD_ = exp( x∙β1+  β2 ∙ ( pmax((x - 1.000)/12.9940517165868, 0)^3 + ((20.815 - 1) ∙ pmax((x - 47.840)/12.9940517165868, 0)^3 - (47.840 - 1) ∙ (pmax((x - 20.815)/12.9940517165868, 0)^3)) / (47.840 - 20.815) ) +  β3*( pmax((x - 9.065)/12.9940517165868, 0)^3 + ((20.815 - 9.065) ∙ pmax((x - 47.840)/12.9940517165868, 0)^3 - (47.840 - 9.065) ∙ (pmax((x - 20.815)/12.9940517165868, 0)^3)) / (47.840 - 20.815) )  β1 = -0.03892253  β2 = 0.20524216  β3 = -0.34804082 | World Health Organization 2023 (8) |
| Epilepsy (ICD-10 coding: G40-41) | Both / All ages | RR_CD_ = exp(β1∙x + 0.5100)  β1 = 1.22861 | Samokhvalov et al., 2010 (9) |
| Hypertensive heart disease (ICD-10 coding: I10-15) | Male / All ages | If(0 ≤ x <10) RR_CD_ = exp(β1∙x)  If(10 ≤ x <30) RR_CD_ = exp(β1*10 + β2∙ (x-10) )  If(30 ≤ x) RR_CD_ = exp(β1*10 + β2*(20) + β3*(x-30) )  β1 = 0.013976194  β2 = 0.00689349  β3 =0.002942025 | Liu et al., 2020 (10) |
|  | Females / All ages | If(0 ≤ x <10) RR_CD_ = exp(β1*x)  If(10 ≤ x <30) RR_CD_ = exp(β1*10 + β2*(x-10) )  If(30 ≤ x) RR_CD_ = exp(β1*10 + β2*(20) + β3*(x-30) )  β1 = 0.005826891  β2 = 0.005362277  β3 =0.005605865 | Liu et al., 2020 (10) |
| Ischaemic heart disease (ICD-10 coding: I20-25) | Males /  15 to 34 years of age | $\mathrm{If}\left( x<60 \right)\mathrm{RR}_{\mathrm{CD}}=exp(\beta_{1}\cdot\left( \beta_{2}\cdot\sqrt{y_{1}}+\beta_{3}\cdot y_{1}^{3} \right))$  $\mathrm{If}\left( 60\leq x<100 \right)\mathrm{RR}_{\mathrm{CD}}=0.04571551+exp(\beta_{1}\cdot\left( \beta_{2}\cdot\sqrt{y_{2}}+\beta_{3}\cdot y_{2}^{3} \right))$  $\mathrm{If}\left( 100\leq x \right)\mathrm{RR}_{\mathrm{CD}}=exp(\beta_{4}\cdot\left( x-100) \right)-1+0.04571551+exp(\beta_{1}\cdot\left( \beta_{2}\cdot\sqrt{y_{2}}+\beta_{3}\cdot y_{2}^{3} \right))$  Where:$y_{1}=\frac{x+0.0099999997764826}{100}$  $y_{2}=\frac{60+0.0099999997764826}{100}$  β1 = 1.111874  β2 = -0.4870068  β3 = 1.550984  β4 = 0.012 | Rehm et al., 2016 (11) based on Roerecke & Rehm, 2012 (12) |
|  | Males /  35 to 64 years of age | $\mathrm{If}\left( x<60 \right)\mathrm{RR}_{\mathrm{CD}}=exp(\beta_{1}\cdot\left( \beta_{2}\cdot\sqrt{y_{1}}+\beta_{3}\cdot y_{1}^{3} \right))$  $\mathrm{If}\left( 60\leq x<100 \right)\mathrm{RR}_{\mathrm{CD}}=0.04571551+exp(\beta_{1}\cdot\left( \beta_{2}\cdot\sqrt{y_{2}}+\beta_{3}\cdot y_{2}^{3} \right))$  $\mathrm{If}\left( 100\leq x \right)\mathrm{RR}_{\mathrm{CD}}=exp(\beta_{4}\cdot\left( x-100) \right)-1+0.04571551+exp(\beta_{1}\cdot\left( \beta_{2}\cdot\sqrt{y_{2}}+\beta_{3}\cdot y_{2}^{3} \right))$  Where:$y_{1}=\frac{x+0.0099999997764826}{100}$  $y_{2}=\frac{60+0.0099999997764826}{100}$  β1 = 0.757104  β2 = -0.4870068  β3 = 1.550984  β4 = 0.012 | Rehm et al., 2016 (11) based on Roerecke & Rehm, 2012 (12) |
|  | Males /  65 years of age and older | $\mathrm{If}\left( x<60 \right)\mathrm{RR}_{\mathrm{CD}}=\exp\left( \beta_{1}\cdot\left( \beta_{2}\cdot\sqrt{y_{1}}+\beta_{3}\cdot y_{1}^{3} \right) \right)$  $\mathrm{If}\left( 60\leq x<100 \right)\mathrm{RR}_{\mathrm{CD}}=0.04571551+exp(\beta_{1}\cdot\left( \beta_{2}\cdot\sqrt{y_{2}}+\beta_{3}\cdot y_{2}^{3} \right))$  $\mathrm{If}\left( 100\leq x \right)\mathrm{RR}_{\mathrm{CD}}=exp(\beta_{4}\cdot\left( x-100) \right)-1+0.04571551+exp(\beta_{1}\cdot\left( \beta_{2}\cdot\sqrt{y_{2}}+\beta_{3}\cdot y_{2}^{3} \right))$  Where $y_{1}=\frac{x+0.0099999997764826}{100}$  $y_{2}=\frac{60+0.0099999997764826}{100}$  β1 = 1.035623  β2 = -0.4870068  β3 = 1.550984  β4 = 0.012 | Rehm et al., 2016 (11) based on Roerecke & Rehm, 2012 (12) |
|  | Females /  15 to 34 years of age | $\mathrm{If}\left( x<30.3814 \right)\mathrm{RR}_{\mathrm{CD}}=exp(\beta_{1}\cdot\left( \beta_{2}\cdot y_{1}+\beta_{3}\cdot y_{1}\cdot ln(y_{2}) \right))$  $\mathrm{If}\left( 30.3814\leq x \right)\mathrm{RR}_{\mathrm{CD}}=exp(\beta_{4}\cdot\left( x-30.3814) \right)-1+\exp\left( \beta_{1}\cdot\left( \beta_{2}\cdot y_{2}+\beta_{3}\cdot y_{2}\cdot ln(y_{2}) \right) \right))$  Where:$y_{1}=\frac{x+0.0099999997764826}{100}$  $y_{2}=\frac{30.3814+0.0099999997764826}{100}$  β1 = 1.111874  β2 = 1.832441  β3 = 1.538557  β4 = 0.01 | Rehm et al., 2016 (11) based on Roerecke & Rehm, 2012 (12) |
|  | Females /  35 to 64 years of age | $\mathrm{If}\left( x<30.3814 \right)\mathrm{RR}_{\mathrm{CD}}=exp(\beta_{1}\cdot\left( \beta_{2}\cdot y_{1}+\beta_{3}\cdot y_{1}\cdot ln(y_{2}) \right))$  $\mathrm{If}\left( 30.3814\leq x \right)\mathrm{RR}_{\mathrm{CD}}=exp(\beta_{4}\cdot\left( x-30.3814) \right)-1+\exp\left( \beta_{1}\cdot\left( \beta_{2}\cdot y_{2}+\beta_{3}\cdot y_{2}\cdot ln(y_{2}) \right) \right))$  Where:$y_{1}=\frac{x+0.0099999997764826}{100}$  $y_{2}=\frac{30.3814+0.0099999997764826}{100}$  β1 = 1.035623  β2 = 1.832441  β3 = 1.538557  β4 = 0.009300 | Rehm et al., 2016 (11) based on Roerecke & Rehm, 2012 (12) |
|  | Females /  65 years of age and older | $\mathrm{If}\left( x<30.3814 \right)\mathrm{RR}_{\mathrm{CD}}=exp(\beta_{1}\cdot\left( \beta_{2}\cdot y_{1}+\beta_{3}\cdot y_{1}\cdot ln(y_{2}) \right))$  $\mathrm{If}\left( 30.3814\leq x \right)\mathrm{RR}_{\mathrm{CD}}=exp(\beta_{4}\cdot\left( x-30.3814) \right)-1+\exp\left( \beta_{1}\cdot\left( \beta_{2}\cdot y_{2}+\beta_{3}\cdot y_{2}\cdot ln(y_{2}) \right) \right))$  Where:$y_{1}=\frac{x+0.0099999997764826}{100}$  $y_{2}=\frac{30.3814+0.0099999997764826}{100}$  β1 = 0.757104  β2 = 1.832441  β3 = 1.538557  β4 = 0.006800 | Rehm et al., 2016 (11) based on Roerecke & Rehm, 2012 (12) |
| Ischaemic stroke (ICD-10 coding: G45–46.8, I63–63.9, I65–66.9, I67.2–67.848, I69.3–69.4) | Males /  15 to 34 years of age | $\mathrm{If}\left( x\leq1 \right)\mathrm{RR}_{\mathrm{CD}}=1-x\cdot(1-exp\left( \beta_{1}\cdot\left( \beta_{2}\cdot\sqrt{y_{1}}+\beta_{3}\cdot\sqrt{y_{1}}\cdot\ln\left( y_{1} \right) \right) \right))$  $\mathrm{If}\left( x>1 \right)\mathrm{RR}_{\mathrm{CD}}=exp(\beta_{1}\cdot\left( \beta_{2}\cdot\sqrt{y_{2}}+\beta_{3}\cdot\sqrt{y_{2}}\cdot ln(y_{2}) \right))$  Where:$y_{1}=\frac{1+0.0028572082519531}{100}$  $y_{2}=\frac{x+0.0028572082519531}{100}$  β1 = 1.111874  β2 = 0.4030081  β3 = 0.3877538 | Rehm et al., 2016 (11) based on Patra et al., 2010 (13) |
|  | Males /  35 to 64 years of age | $\mathrm{If}\left( x\leq1 \right)\mathrm{RR}_{\mathrm{CD}}=1-x\cdot(1-exp\left( \beta_{1}\cdot\left( \beta_{2}\cdot\sqrt{y_{1}}+\beta_{3}\cdot\sqrt{y_{1}}\cdot\ln\left( y_{1} \right) \right) \right))$  $\mathrm{If}\left( x>1 \right)\mathrm{RR}_{\mathrm{CD}}=exp(\beta_{1}\cdot\left( \beta_{2}\cdot\sqrt{y_{2}}+\beta_{3}\cdot\sqrt{y_{2}}\cdot ln(y_{2}) \right))$  Where $y_{1}=\frac{1+0.0028572082519531}{100}$  $y_{2}=\frac{x+0.0028572082519531}{100}$  β1 = 1.035623  β2 = 0.4030081  β3 = 0.3877538 | Rehm et al., 2016 (11) based on Patra et al., 2010 (13) |
|  | Males /  65 years of age and older | $\mathrm{If}\left( x\leq1 \right)\mathrm{RR}_{\mathrm{CD}}=1-x\cdot(1-exp\left( \beta_{1}\cdot\left( \beta_{2}\cdot\sqrt{y_{1}}+\beta_{3}\cdot\sqrt{y_{1}}\cdot\ln\left( y_{1} \right) \right) \right))$  $\mathrm{If}\left( x>1 \right)\mathrm{RR}_{\mathrm{CD}}=exp(\beta_{1}\cdot\left( \beta_{2}\cdot\sqrt{y_{2}}+\beta_{3}\cdot\sqrt{y_{2}}\cdot ln(y_{2}) \right))$  Where$y_{1}=\frac{1+0.0028572082519531}{100}$  $y_{2}=\frac{x+0.0028572082519531}{100}$  β1 = 0.757104  β2 = 0.4030081  β3 = 0.3877538 | Rehm et al., 2016 (11) based on Patra et al., 2010 (13) |
|  | Females /  15 to 34 years of age | $\mathrm{If}\left( x\leq1 \right)\mathrm{RR}_{\mathrm{CD}}=1-x\cdot(1-exp\left( \beta_{2}\cdot\sqrt{y_{1}}+\beta_{3}\cdot y_{1} \right)$  $\mathrm{If}\left( x>1 \right)\mathrm{RR}_{\mathrm{CD}}=exp(\beta_{1}\cdot\left( \beta_{2}\cdot\sqrt{y_{2}}+\beta_{3}\cdot y_{2} \right))$  Where:$y_{1}=\frac{1+0.0028572082519531}{100}$  $y_{2}=\frac{x+0.0028572082519531}{100}$  β1 = 1.111874  β2 = -2.48768  β3 = 3.7087240 | Rehm et al., 2016 (11) based on Patra et al., 2010 (13) |
|  | Females /  35 to 64 years of age | $\mathrm{If}\left( x\leq1 \right)\mathrm{RR}_{\mathrm{CD}}=1-x\cdot(1-exp\left( \beta_{2}\cdot\sqrt{y_{1}}+\beta_{3}\cdot y_{1} \right)$  $\mathrm{If}\left( x>1 \right)\mathrm{RR}_{\mathrm{CD}}=exp(\beta_{1}\cdot\left( \beta_{2}\cdot\sqrt{y_{2}}+\beta_{3}\cdot y_{2} \right))$  Where:$y_{1}=\frac{1+0.0028572082519531}{100}$  $y_{2}=\frac{x+0.0028572082519531}{100}$  β1 = 1.035623  β2 = -2.48768  β3 = 3.7087240 | Rehm et al., 2016 (11) based on Patra et al., 2010 (13) |
|  | Females /  65 years of age and older | $\mathrm{If}\left( x\leq1 \right)\mathrm{RR}_{\mathrm{CD}}=1-x\cdot(1-exp\left( \beta_{2}\cdot\sqrt{y_{1}}+\beta_{3}\cdot y_{1} \right)$  $\mathrm{If}\left( x>1 \right)\mathrm{RR}_{\mathrm{CD}}=exp(\beta_{1}\cdot\left( \beta_{2}\cdot\sqrt{y_{2}}+\beta_{3}\cdot y_{2} \right))$  Where:$y_{1}=\frac{1+0.0028572082519531}{100}$  $y_{2}=\frac{x+0.0028572082519531}{100}$  β1 = 0.757104  β2 = -2.48768  β3 = 3.7087240 | Rehm et al., 2016 (11) based on Patra et al., 2010 (13) |
| Haemorrhagic stroke (ICD-10 coding: I60–62.9, I67.0–67.1, I69.0–69.298) | Males / All Ages | $\mathrm{If}\left( x\leq1 \right)\mathrm{RR}_{\mathrm{CD}}=1-x\cdot(1-exp\left( \beta_{1}\cdot\frac{(1+0.0028572082519531)}{100} \right)$  $\mathrm{If}\left( x>1 \right)\mathrm{RR}_{\mathrm{CD}}=exp(\beta_{1}\cdot\frac{(1+0.0028572082519531)}{100})$  β1 = 0.6898937 | Larsson et al., 2016 (14) |
|  | Females / All Ages | $\mathrm{If}\left( x\leq1 \right)\mathrm{RR}_{\mathrm{CD}}=1-x\cdot(1-exp\left( \beta_{1}\cdot\frac{(1+0.0028572082519531)}{100} \right)$  $\mathrm{If}\left( x>1 \right)\mathrm{RR}_{\mathrm{CD}}=exp(\beta_{1}\cdot\frac{(1+0.0028572082519531)}{100})$  β1 = 1.466406 | Larsson et al., 2016 (14) |
| Atrial fibrillation and flutter (ICD-10: I48) | Both / All Ages | RR_CD_ = exp(x∙β1)  β1 = 0.00641342 | Larsson et al., 2014 (15) |
| Cirrhosis (ICD-10 coding: K70, K74) | Males / All Ages | $\mathrm{If}\left( x\leq1 \right)\mathrm{RR}_{\mathrm{CD}}=1+x\cdot(exp\left( \left( \beta_{1}+\beta_{2} \right)\cdot\frac{\left( 1+0.1699981689453125 \right)}{100}-1 \right)$  $\mathrm{If}\left( x>1 \right)\mathrm{RR}_{\mathrm{CD}}=exp\left( \left( \beta_{1}+\beta_{2} \right)\cdot\frac{\left( 1+0.1699981689453125 \right)}{100} \right)$  β1 = 1.687111  β2 = 1.106413 | Roerecke et al., 2019 (16) |
|  | Females / All Ages | $\mathrm{If}\left( x\leq1 \right)\mathrm{RR}_{\mathrm{CD}}=1+x\cdot(exp\left( \left( \beta_{1}+\beta_{2} \right)\cdot\sqrt{\frac{\left( 1+0.1699981689453125 \right)}{100}}-1 \right)$  $\mathrm{If}\left( x>1 \right)\mathrm{RR}_{\mathrm{CD}}=exp\left( \left( \beta_{1}+\beta_{2} \right)\cdot\sqrt{\frac{\left( 1+0.1699981689453125 \right)}{100}} \right)$  β1 = 2.351821  β2 = 0.9002139 | Roerecke et al., 2019 (16) |
| Pancreatitis (ICD-10 coding: K85-86) | Males / All Ages | RR_CD_ = exp(x∙β1)  β1 = 0.0173451 | Samakvalov et al., 2015 (17) |
|  | Females / All Ages | $\mathrm{If}\left( x<3 \right)\mathrm{RR}_{\mathrm{CD}}=exp(\beta_{1}\cdot x)$  $\mathrm{If}\left( 3\leq x<15 \right)\mathrm{RR}_{\mathrm{CD}}=exp(\beta_{1}\cdot x+\beta_{2}\cdot\frac{{(x-3)}^{3}}{{(40-3)}^{2}})$  $\mathrm{If}\left( 15\leq x<40 \right)\mathrm{RR}_{\mathrm{CD}}=exp(\beta_{1}\cdot x+\beta_{2}\cdot\frac{{(x-3)}^{3}-\frac{{(x-15)}^{3}*(40-3)}{(40-15)}}{{(40-3)}^{2}})$  $\mathrm{If}\left( 40\leq x<108 \right)\mathrm{RR}_{\mathrm{CD}}=exp(\beta_{1}\cdot x+\beta_{2}\cdot\frac{{(x-3)}^{3}-\frac{{(x-15)}^{3}*\left( 40-3 \right)-{(x-40)}^{3}*(15-3)}{(40-15)}}{{(40-3)}^{2}})$  $\mathrm{If}\left( x>108 \right)\mathrm{RR}_{\mathrm{CD}}=\exp\left( \beta_{1}\cdot108+\beta_{2}\cdot\frac{\left( 108-3 \right)^{3}-\frac{\left( 108-15 \right)^{3}*\left( 40-3 \right)-\left( 108-40 \right)^{3}*\left( 15-3 \right)}{\left( 40-15 \right)}}{\left( 40-3 \right)^{2}} \right)$  β1 = -0.0272886  β2 = 0.0611466 | Samakvalov et al., 2015 (17) |
| Road injuries (ICD-10 coding: V01-04, V06, V09-80, V87, V89, V99) | Both  (Non-heavy episodic drinkers) / All Ages | $\mathrm{RR}_{\mathrm{CD}}=exp(\beta_{1}\cdot x)$  β1 = 0.00299550897979837 | World Health Organization 2018 (18) |
|  | Both  (Heavy episodic drinkers) / All Ages | $\mathrm{RR}_{\mathrm{CD}}=exp(\beta_{1}\cdot x+\beta_{2})$  β1 = 0.00299550897979837  β2 = 0.959350221334602 | World Health Organization 2018 (18) |
| Unintentional injuries (ICD-10 coding: V01-X40, X43, X46-59, Y40-86, Y88, Y89 (excluding road injuries)) | Both  (Non-heavy episodic drinkers) / All Ages | $\mathrm{RR}_{\mathrm{CD}}=exp(\beta_{1}\cdot x)$  β1 = 0.00199800266267306 | World Health Organization 2018 (18) |
|  | Both  (Heavy episodic drinkers) / All Ages | $\mathrm{RR}_{\mathrm{CD}}=exp(\beta_{1}\cdot x+\beta_{2})$  β1 = 0.00199800266267306  β2 = 0.647103242058538 | World Health Organization 2018 (18) |
| Intentional injuries (ICD-10 coding: X60-Y09, Y35-36, Y870, Y871) | Both  (Non-heavy episodic drinkers) / All Ages | $\mathrm{RR}_{\mathrm{CD}}=exp(\beta_{1}\cdot x)$  β1 = 0.00199800266267306 | World Health Organization 2018 (18) |
|  | Both  (Heavy episodic drinkers) / All Ages | $\mathrm{RR}_{\mathrm{CD}}=exp(\beta_{1}\cdot x+\beta_{2})$  β1 = 0.00199800266267306  β2 = 0.647103242058538 | World Health Organization 2018 (18) |

The pmax function compares the values of the two vectors in pairs and returns a final vector that contains the maximum values from each pair. This function is used in restricted cubic splines.

### Table AF2: Relative Risks for partially attributable disease categories for Russia and similar countries

| Disease | Sex / Age | Relative risk function (where x is the daily alcohol consumption in g) | Source |
| --- | --- | --- | --- |
| Tuberculosis (ICD-10 codes: A15-19, B90) | Males / All Ages | $\mathrm{If}\left( x<25.36 \right)\mathrm{RR}_{\mathrm{CD}}=exp(\beta_{1})$  $\mathrm{If}\left( 25.36\leq x<76.08 \right)\mathrm{RR}_{\mathrm{CD}}=exp(\beta_{2})$  $\mathrm{If}\left( 76.08\leq x \right)\mathrm{RR}_{\mathrm{CD}}=exp(\beta_{3})$  β1 = ln(1.01)  β2 = ln(1.97)  β3 = ln(4.14) | Zaridze et al., 2009 (19, 20) |
|  | Females / All Ages | $\mathrm{If}\left( x<25.36 \right)\mathrm{RR}_{\mathrm{CD}}=exp(\beta_{1})$  $\mathrm{If}\left( 25.36\leq x<76.08 \right)\mathrm{RR}_{\mathrm{CD}}=exp(\beta_{2})$  $\mathrm{If}\left( 76.08\leq x \right)\mathrm{RR}_{\mathrm{CD}}=exp(\beta_{3})$  β1 = ln(0.93)  β2 = ln(4.06)  β3 = ln(5.32) | Zaridze et al., 2009 (19, 20) |
| Lower respiratory infections (ICD-10 codes: J09-22, P23, U04) | Males / All Ages | $\mathrm{If}\left( x<25.36 \right)\mathrm{RR}_{\mathrm{CD}}=exp(\beta_{1})$  $\mathrm{If}\left( 25.36\leq x<76.08 \right)\mathrm{RR}_{\mathrm{CD}}=exp(\beta_{2})$  $\mathrm{If}\left( 76.08\leq x \right)\mathrm{RR}_{\mathrm{CD}}=exp(\beta_{3})$  β1 = ln(0.95)  β2 = ln(1.92)  β3 = ln(3.29) | Zaridze et al., 2009 (19, 20) |
|  | Females / All Ages | $\mathrm{If}\left( x<25.36 \right)\mathrm{RR}_{\mathrm{CD}}=exp(\beta_{1})$  $\mathrm{If}\left( 25.36\leq x<76.08 \right)\mathrm{RR}_{\mathrm{CD}}=exp(\beta_{2})$  $\mathrm{If}\left( 76.08\leq x \right)\mathrm{RR}_{\mathrm{CD}}=exp(\beta_{3})$  β1 = ln(2.10)  β2 = ln(3.21)  β3 = ln(3.42) | Zaridze et al., 2009 (19, 20) |
| Ischaemic heart disease (ICD-10 codes: I20-25) | Males / All Ages | $\mathrm{If}\left( x<25.36 \right)\mathrm{RR}_{\mathrm{CD}}=exp(\beta_{1})$  $\mathrm{If}\left( 25.36\leq x<76.08 \right)\mathrm{RR}_{\mathrm{CD}}=exp(\beta_{2})$  $\mathrm{If}\left( 76.08\leq x \right)\mathrm{RR}_{\mathrm{CD}}=exp(\beta_{3})$  β1 = ln(1.09178)  β2 = ln(1.49618)  β3 = ln(2.43944) | Zaridze et al., 2009 (19, 20) |
|  | Females / All Ages | $\mathrm{If}\left( x<25.36 \right)\mathrm{RR}_{\mathrm{CD}}=exp(\beta_{1})$  $\mathrm{If}\left( 25.36\leq x<76.08 \right)\mathrm{RR}_{\mathrm{CD}}=exp(\beta_{2})$  $\mathrm{If}\left( 76.08\leq x \right)\mathrm{RR}_{\mathrm{CD}}=exp(\beta_{3})$  β1 = ln(1.51383)  β2 = ln(3.43525)  β3 = ln(7.41902) | Zaridze et al., 2009 (19, 20) |
| Ischaemic stroke (ICD-10 codes: G45–46.8, I63–63.9, I65–66.9, I67.2–67.848, I69.3–69.4) | Males / All Ages | $\mathrm{If}\left( x<25.36 \right)\mathrm{RR}_{\mathrm{CD}}=exp(\beta_{1})$  $\mathrm{If}\left( 25.36\leq x<76.08 \right)\mathrm{RR}_{\mathrm{CD}}=exp(\beta_{2})$  $\mathrm{If}\left( 76.08\leq x \right)\mathrm{RR}_{\mathrm{CD}}=exp(\beta_{3})$  β1 = ln(1.06)  β2 = ln(1.14)  β3 = ln(1.28) | Zaridze et al., 2009 (19, 20) |
|  | Females / All Ages | $\mathrm{If}\left( x<25.36 \right)\mathrm{RR}_{\mathrm{CD}}=exp(\beta_{1})$  $\mathrm{If}\left( 25.36\leq x<76.08 \right)\mathrm{RR}_{\mathrm{CD}}=exp(\beta_{2})$  $\mathrm{If}\left( 76.08\leq x \right)\mathrm{RR}_{\mathrm{CD}}=exp(\beta_{3})$  β1 = ln(1.38)  β2 = ln(1.36)  β3 = ln(1.62) | Zaridze et al., 2009 (19, 20) |
| Haemorrhagic stroke (ICD-10 codes: I60–62.9, I67.0–67.1, I69.0–69.298) | Males / All Ages | $\mathrm{If}\left( x<25.36 \right)\mathrm{RR}_{\mathrm{CD}}=exp(\beta_{1})$  $\mathrm{If}\left( 25.36\leq x<76.08 \right)\mathrm{RR}_{\mathrm{CD}}=exp(\beta_{2})$  $\mathrm{If}\left( 76.08\leq x \right)\mathrm{RR}_{\mathrm{CD}}=exp(\beta_{3})$  β1 = ln(1.06)  β2 = ln(1.14)  β3 = ln(1.28) | Zaridze et al., 2009 (19, 20) |
|  | Females / All Ages | $\mathrm{If}\left( x<25.36 \right)\mathrm{RR}_{\mathrm{CD}}=exp(\beta_{1})$  $\mathrm{If}\left( 25.36\leq x<76.08 \right)\mathrm{RR}_{\mathrm{CD}}=exp(\beta_{2})$  $\mathrm{If}\left( 76.08\leq x \right)\mathrm{RR}_{\mathrm{CD}}=exp(\beta_{3})$  β1 = ln(1.38)  β2 = ln(1.36)  β3 = ln(1.62) | Zaridze et al., 2009 (19, 20) |
| Cirrhosis (ICD-10 codes : K70, K74) | Males / All Ages | $\mathrm{If}\left( x<25.36 \right)\mathrm{RR}_{\mathrm{CD}}=exp(\beta_{1})$  $\mathrm{If}\left( 25.36\leq x<76.08 \right)\mathrm{RR}_{\mathrm{CD}}=exp(\beta_{2})$  $\mathrm{If}\left( 76.08\leq x \right)\mathrm{RR}_{\mathrm{CD}}=exp(\beta_{3})$  β1 = ln(0.92)  β2 = ln(1.77)  β3 = ln(6.21) | Zaridze et al., 2009 (19, 20) |
|  | Females / All Ages | $\mathrm{If}\left( x<25.36 \right)\mathrm{RR}_{\mathrm{CD}}=exp(\beta_{1})$  $\mathrm{If}\left( 25.36\leq x<76.08 \right)\mathrm{RR}_{\mathrm{CD}}=exp(\beta_{2})$  $\mathrm{If}\left( 76.08\leq x \right)\mathrm{RR}_{\mathrm{CD}}=exp(\beta_{3})$  β1 = ln(2.50)  β2 = ln(7.07)  β3 = ln(12.08) | Zaridze et al., 2009 (19, 20) |
| Pancreatitis (ICD-10 codes: K85-86) | Males / All Ages | $\mathrm{If}\left( x<25.36 \right)\mathrm{RR}_{\mathrm{CD}}=exp(\beta_{1})$  $\mathrm{If}\left( 25.36\leq x<76.08 \right)\mathrm{RR}_{\mathrm{CD}}=exp(\beta_{2})$  $\mathrm{If}\left( 76.08\leq x \right)\mathrm{RR}_{\mathrm{CD}}=exp(\beta_{3})$  β1 = ln(1.43)  β2 = ln(2.07)  β3 = ln( 6.69) | Zaridze et al., 2009 (19, 20) |
|  | Females / All Ages | $\mathrm{If}\left( x<25.36 \right)\mathrm{RR}_{\mathrm{CD}}=exp(\beta_{1})$  $\mathrm{If}\left( 25.36\leq x<76.08 \right)\mathrm{RR}_{\mathrm{CD}}=exp(\beta_{2})$  $\mathrm{If}\left( 76.08\leq x \right)\mathrm{RR}_{\mathrm{CD}}=exp(\beta_{3})$  β1 = ln(1.09)  β2 = ln(5.01)  β3 = ln(19.26) | Zaridze et al., 2009 (19, 20) |
| Road injury (ICD-10 codes: V01–04, V06, V09–80, V87, V89, V99) | Males / All Ages | $\mathrm{If}\left( x<25.36 \right)\mathrm{RR}_{\mathrm{CD}}=exp(\beta_{1})$  $\mathrm{If}\left( 25.36\leq x<76.08 \right)\mathrm{RR}_{\mathrm{CD}}=exp(\beta_{2})$  $\mathrm{If}\left( 76.08\leq x \right)\mathrm{RR}_{\mathrm{CD}}=exp(\beta_{3})$  β1 = ln(1.52)  β2 = ln(2.68)  β3 = ln(4.20) | Zaridze et al., 2009 (19, 20) |
|  | Females / All Ages | $\mathrm{If}\left( x<25.36 \right)\mathrm{RR}_{\mathrm{CD}}=exp(\beta_{1})$  $\mathrm{If}\left( 25.36\leq x<76.08 \right)\mathrm{RR}_{\mathrm{CD}}=exp(\beta_{2})$  $\mathrm{If}\left( 76.08\leq x \right)\mathrm{RR}_{\mathrm{CD}}=exp(\beta_{3})$  β1 = ln(1.98)  β2 = ln(4.48)  β3 = ln(3.17) | Zaridze et al., 2009 (19, 20) |
| Other unintentional injuries (ICD 10 codes: X40, X43, X46–48, X49, W00–19, X00–19, W65–74, W20–38, W40–43, W45, W46, W49–52, W75, W76, Rest of V, W39, W44, W53–64, W77–99, X20–29, X50–59, Y40–86, Y88, Y89) | Males / All Ages | $\mathrm{If}\left( x<25.36 \right)\mathrm{RR}_{\mathrm{CD}}=exp(\beta_{1})$  $\mathrm{If}\left( 25.36\leq x<76.08 \right)\mathrm{RR}_{\mathrm{CD}}=exp(\beta_{2})$  $\mathrm{If}\left( 76.08\leq x \right)\mathrm{RR}_{\mathrm{CD}}=exp(\beta_{3})$  β1 = ln(1.58)  β2 = ln(2.48)  β3 = ln(6.07) | Zaridze et al., 2009 (19, 20) |
|  | Females / All Ages | $\mathrm{If}\left( x<25.36 \right)\mathrm{RR}_{\mathrm{CD}}=exp(\beta_{1})$  $\mathrm{If}\left( 25.36\leq x<76.08 \right)\mathrm{RR}_{\mathrm{CD}}=exp(\beta_{2})$  $\mathrm{If}\left( 76.08\leq x \right)\mathrm{RR}_{\mathrm{CD}}=exp(\beta_{3})$  β1 = ln(2.08)  β2 = ln(5.24)  β3 = ln(8.56) | Zaridze et al., 2009 (19, 20) |
| Self-harm (ICD 10 codes: X60–84, Y870) | Males / All Ages | $\mathrm{If}\left( x<25.36 \right)\mathrm{RR}_{\mathrm{CD}}=exp(\beta_{1})$  $\mathrm{If}\left( 25.36\leq x<76.08 \right)\mathrm{RR}_{\mathrm{CD}}=exp(\beta_{2})$  $\mathrm{If}\left( 76.08\leq x \right)\mathrm{RR}_{\mathrm{CD}}=exp(\beta_{3})$  β1 = ln(1.21)  β2 = ln(3.47)  β3 = ln(8.62) | Zaridze et al., 2009 (19, 20) |
|  | Females / All Ages | $\mathrm{If}\left( x<25.36 \right)\mathrm{RR}_{\mathrm{CD}}=exp(\beta_{1})$  $\mathrm{If}\left( 25.36\leq x<76.08 \right)\mathrm{RR}_{\mathrm{CD}}=exp(\beta_{2})$  $\mathrm{If}\left( 76.08\leq x \right)\mathrm{RR}_{\mathrm{CD}}=exp(\beta_{3})$  β1 = ln(2.82)  β2 = ln(8.22)  β3 = ln(14.75) | Zaridze et al., 2009 (19, 20) |
| Interpersonal violence (ICD 10 codes: X85–Y09, Y871) | Males / All Ages | $\mathrm{If}\left( x<25.36 \right)\mathrm{RR}_{\mathrm{CD}}=exp(\beta_{1})$  $\mathrm{If}\left( 25.36\leq x<76.08 \right)\mathrm{RR}_{\mathrm{CD}}=exp(\beta_{2})$  $\mathrm{If}\left( 76.08\leq x \right)\mathrm{RR}_{\mathrm{CD}}=exp(\beta_{3})$  β1 = ln(1.75)  β2 = ln(3.67)  β3 = ln(9.47) | Zaridze et al., 2009 (19, 20) |
|  | Females / All Ages | $\mathrm{If}\left( x<25.36 \right)\mathrm{RR}_{\mathrm{CD}}=exp(\beta_{1})$  $\mathrm{If}\left( 25.36\leq x<76.08 \right)\mathrm{RR}_{\mathrm{CD}}=exp(\beta_{2})$  $\mathrm{If}\left( 76.08\leq x \right)\mathrm{RR}_{\mathrm{CD}}=exp(\beta_{3})$  β1 = ln(3.55)  β2 = ln(10.23)  β3 = ln(19.11) | Zaridze et al., 2009 (19, 20) |

### References

1. Imtiaz S., Shield K. D., Roerecke M., Samokhvalov A. V., Lönnroth K., Rehm J. Alcohol consumption as a risk factor for tuberculosis: meta-analyses and burden of disease, Eur Respir J 2017: 50: 1700216.

2. Rehm J., Probst C., Shield K. D., Shuper P. A. Does alcohol use have a causal effect on HIV incidence and disease progression? A review of the literature and a modeling strategy for quantifying the effect, Popul Health Metr 2017: 15: 4.

3. Samokhvalov A., Irving H., Rehm J. Alcohol consumption as a risk factor for pneumonia: a systematic review and meta-analysis, Epidemiol Infect 2010: 138: 1789-1795.

4. Bagnardi V., Rota M., Botteri E., Tramacere I., Islami F., Fedirko V. et al. Alcohol consumption and site-specific cancer risk: a comprehensive dose-response meta-analysis, Br J Cancer 2015: 112: 580-593.

5. Vieira A., Abar L., Chan D., Vingeliene S., Polemiti E., Stevens C. et al. Foods and beverages and colorectal cancer risk: a systematic review and meta-analysis of cohort studies, an update of the evidence of the WCRF-AICR Continuous Update Project, Annals of Oncology 2017: 28: 1788-1802.

6. World Cancer Research Fund/American Institute for Cancer Research. Diet, nutrition, physical activity and cancer: a global perspective. Continuous Update Project expert report 2018. , London, United Kingdom; 2018.

7. Sun Q., Xie W., Wang Y., Chong F., Song M., Li T. et al. Alcohol consumption by beverage type and risk of breast cancer: a dose-response meta-analysis of prospective cohort studies, Alcohol and Alcoholism 2020: 55: 246-253.

8. World Health Organization. Global Information System on Alcohol and Health, Geneva, Switzerland: World Health Organization; 2023.

9. Samokhvalov A. V., Irving H., Mohapatra S., Rehm J. Alcohol consumption, unprovoked seizures, and epilepsy: A systematic review and meta‐analysis, Epilepsia 2010: 51: 1177-1184.

10. Liu F., Liu Y., Sun X., Yin Z., Li H., Deng K. et al. Race-and sex-specific association between alcohol consumption and hypertension in 22 cohort studies: A systematic review and meta-analysis, Nutrition, Metabolism and Cardiovascular Diseases 2020: 30: 1249-1259.

11. Rehm J., Shield K. D., Roerecke M., Gmel G. Modelling the impact of alcohol consumption on cardiovascular disease mortality for comparative risk assessments: an overview BMC Public Health 2016: 16: 363.

12. Roerecke M., Rehm J. The cardioprotective association of average alcohol consumption and ischaemic heart disease: a systematic review and meta-analysis, Addiction 2012: 107: 1246-1260.

13. Patra J., Taylor B., Irving H., Roerecke M., Baliunas D., Mohapatra S. et al. Alcohol consumption and the risk of morbidity and mortality from different stroke types - a systematic review and meta-analysis, BMC Public Health 2010: 10: 258.

14. Larsson S. C., Wallin A., Wolk A., Markus H. S. Differing association of alcohol consumption with different stroke types: a systematic review and meta-analysis, BMC medicine 2016: 14: 1-11.

15. Larsson S. C., Drca N., Wolk A. Alcohol consumption and risk of atrial fibrillation: a prospective study and dose-response meta-analysis, Journal of the American College of Cardiology 2014: 64: 281-289.

16. Roerecke M., Vafaei A., Hasan O. S. M., Chrystoja B. R., Cruz M., Lee R. et al. Alcohol consumption and risk of liver cirrhosis: a systematic review and meta-analysis, Am J Gastroenterol 2019: 114: 1574-1586.

17. Samokhvalov A. V., Rehm J., Roerecke M. Alcohol consumption as a risk factor for acute and chronic pancreatitis: a systematic review and a series of meta-analyses, EBioMedicine 2015: 2: 1996-2002.

18. World Health Organization. Global status report on alcohol and health 2018., Geneva, Switzerland: World Health Organization; 2018.

19. Zaridze D., Brennan P., Boreham J., Boroda A., Karpov R., Lazarev A. et al. Alcohol and cause-specific mortality in Russia: a retrospective case–control study of 48 557 adult deaths, Lancet 2009: 373: 2201-2214.

20. Shield K. D., Rehm J. Russia-specific relative risks and their effects on the estimated alcohol-attributable burden of disease, BMC Public Health 2015: 15.

[Additional File 2: Parameters for the alcohol-attributable fraction models](#_Toc150497457) by year

|  | **Lifetime abstainer** | **Current drinker** | **Former drinkers** | **APC in litres pure alcohol** | **APC in grams per day** | **Year** | **Sex** |
| --- | --- | --- | --- | --- | --- | --- | --- |
|  | 14.94 (14.09-15.77) | 67.49 (66.59-68.46) | 17.58 (16.24-18.85) | 4.35 (3.48-5.11) | 13.92 (11.11-16.31) | 2001 | female |
|  | 6.34 (5.94-6.75) | 81.69 (81.02-82.33) | 11.97 (11.23-12.8) | 15.1 (11.91-17.97) | 39.95 (31.51-47.54) | 2001 | male |
|  | 14.35 (13.59-15.12) | 68.12 (67.16-69.04) | 17.54 (16.29-18.73) | 4.63 (3.74-5.36) | 14.7 (11.88-17.08) | 2002 | female |
|  | 6.06 (5.67-6.43) | 82.05 (81.45-82.61) | 11.89 (11.2-12.59) | 16.08 (12.97-18.87) | 42.35 (34.13-49.77) | 2002 | male |
|  | 13.99 (13.27-14.74) | 68.36 (67.49-69.27) | 17.65 (16.46-18.87) | 4.73 (3.86-5.49) | 14.95 (12.16-17.34) | 2003 | female |
|  | 5.89 (5.54-6.23) | 82.16 (81.57-82.77) | 11.95 (11.3-12.66) | 16.41 (13.34-19.36) | 43.16 (35.15-50.95) | 2003 | male |
|  | 13.48 (12.75-14.17) | 68.92 (68.03-69.75) | 17.6 (16.51-18.68) | 5.01 (4.09-5.77) | 15.72 (12.8-18.14) | 2004 | female |
|  | 5.64 (5.3-5.98) | 82.5 (81.88-83.12) | 11.86 (11.15-12.54) | 17.4 (14.15-20.53) | 45.59 (37.07-53.76) | 2004 | male |
|  | 13.15 (12.46-13.84) | 69.15 (68.26-70) | 17.7 (16.53-18.77) | 5.12 (4.21-5.82) | 16.01 (13.13-18.21) | 2005 | female |
|  | 5.47 (5.15-5.8) | 82.64 (82.08-83.22) | 11.9 (11.26-12.53) | 17.79 (14.62-20.78) | 46.53 (38.09-54.2) | 2005 | male |
|  | 12.53 (11.89-13.2) | 69.77 (68.89-70.65) | 17.71 (16.67-18.82) | 5.44 (4.49-6.27) | 16.86 (13.92-19.34) | 2006 | female |
|  | 5.17 (4.88-5.48) | 83 (82.47-83.61) | 11.83 (11.13-12.44) | 18.9 (15.78-22.07) | 49.22 (41.01-57.49) | 2006 | male |
|  | 12.04 (11.4-12.7) | 70.1 (69.15-71.02) | 17.86 (16.75-18.91) | 5.6 (4.65-6.34) | 17.25 (14.38-19.59) | 2007 | female |
|  | 4.93 (4.65-5.19) | 83.19 (82.65-83.7) | 11.88 (11.26-12.46) | 19.44 (16-22.5) | 50.5 (41.5-58.42) | 2007 | male |
|  | 11.85 (11.24-12.52) | 70.11 (69.14-71.03) | 18.04 (16.89-19.2) | 5.58 (4.61-6.31) | 17.2 (14.22-19.51) | 2008 | female |
|  | 4.82 (4.51-5.09) | 83.21 (82.64-83.76) | 11.97 (11.32-12.61) | 19.41 (16.2-22.33) | 50.42 (42.2-57.97) | 2008 | male |
|  | 12.54 (11.89-13.21) | 69.37 (68.42-70.28) | 18.09 (16.93-19.2) | 5.26 (4.37-5.96) | 16.4 (13.62-18.53) | 2009 | female |
|  | 5.09 (4.81-5.39) | 82.87 (82.27-83.43) | 12.04 (11.4-12.69) | 18.41 (15.35-21.34) | 48.01 (40.01-55.48) | 2009 | male |
|  | 12.68 (11.92-13.35) | 68.81 (67.97-69.73) | 18.51 (17.42-19.61) | 4.9 (4.06-5.6) | 15.4 (12.73-17.57) | 2010 | female |
|  | 5.11 (4.8-5.4) | 82.56 (82.01-83.13) | 12.33 (11.69-12.97) | 17.19 (14.08-20.28) | 45 (36.72-53.14) | 2010 | male |
|  | 11.31 (10.71-11.91) | 70.53 (69.57-71.47) | 18.16 (17.01-19.33) | 5.88 (4.93-6.6) | 18.01 (15.08-20.26) | 2011 | female |
|  | 4.45 (4.2-4.69) | 83.66 (83.1-84.21) | 11.9 (11.28-12.5) | 20.64 (17.25-23.75) | 53.33 (44.55-61.29) | 2011 | male |
|  | 11.01 (10.41-11.63) | 70.69 (69.72-71.62) | 18.3 (17.18-19.36) | 5.93 (5.01-6.72) | 18.13 (15.4-20.47) | 2012 | female |
|  | 4.27 (4.02-4.51) | 83.81 (83.22-84.42) | 11.92 (11.26-12.55) | 20.87 (17.45-24.12) | 53.84 (44.97-62.13) | 2012 | male |
|  | 10.77 (10.2-11.3) | 70.72 (69.79-71.67) | 18.51 (17.43-19.61) | 5.88 (4.92-6.61) | 17.98 (15.11-20.16) | 2013 | female |
|  | 4.12 (3.89-4.35) | 83.88 (83.3-84.46) | 12 (11.42-12.63) | 20.76 (17.81-23.85) | 53.51 (45.93-61.52) | 2013 | male |
|  | 10.61 (10.04-11.2) | 70.71 (69.78-71.66) | 18.68 (17.61-19.81) | 5.85 (4.91-6.68) | 17.88 (15.03-20.39) | 2014 | female |
|  | 4.02 (3.8-4.24) | 83.91 (83.36-84.45) | 12.07 (11.5-12.66) | 20.7 (17.52-23.52) | 53.32 (44.95-60.68) | 2014 | male |
|  | 11.11 (10.54-11.7) | 69.66 (68.73-70.65) | 19.22 (18.04-20.26) | 5.15 (4.25-5.85) | 15.99 (13.12-18.12) | 2015 | female |
|  | 4.2 (3.98-4.43) | 83.33 (82.75-83.88) | 12.48 (11.89-13.11) | 18.3 (15.25-21.51) | 47.47 (39.58-55.8) | 2015 | male |
|  | 11.09 (10.47-11.67) | 69.3 (68.41-70.21) | 19.6 (18.56-20.74) | 4.9 (3.99-5.63) | 15.29 (12.48-17.57) | 2016 | female |
|  | 4.15 (3.95-4.38) | 83.14 (82.6-83.68) | 12.71 (12.09-13.29) | 17.46 (14.44-20.38) | 45.4 (37.51-53.08) | 2016 | male |
|  | 11.23 (10.62-11.88) | 68.57 (67.56-69.53) | 20.2 (19.06-21.3) | 4.46 (3.61-5.1) | 14.07 (11.36-16.17) | 2017 | female |
|  | 4.18 (3.96-4.41) | 82.71 (82.11-83.32) | 13.11 (12.48-13.74) | 15.95 (13.09-18.81) | 41.69 (34.26-49.27) | 2017 | male |
|  | 11.16 (10.44-11.81) | 68.22 (67.15-69.24) | 20.62 (19.41-21.9) | 4.25 (3.46-4.94) | 13.45 (10.9-15.74) | 2018 | female |
|  | 4.12 (3.88-4.34) | 82.51 (81.89-83.13) | 13.37 (12.73-14.03) | 15.23 (12.24-17.87) | 39.89 (32.03-46.8) | 2018 | male |
|  | 10.83 (10.19-11.46) | 68.36 (67.4-69.39) | 20.81 (19.51-21.97) | 4.28 (3.47-4.93) | 13.52 (10.96-15.57) | 2019 | female |
|  | 3.95 (3.72-4.17) | 82.6 (82.01-83.24) | 13.45 (12.81-14.07) | 15.37 (12.27-17.9) | 40.22 (32.17-46.67) | 2019 | male |
|  | 10.61 (10.01-11.25) | 68.61 (67.64-69.56) | 20.78 (19.62-21.93) | 4.41 (3.58-5.1) | 13.89 (11.26-16.03) | 2020 | female |
|  | 3.83 (3.61-4.06) | 82.8 (82.15-83.43) | 13.37 (12.73-14.03) | 15.89 (12.99-18.99) | 41.48 (33.95-49.32) | 2020 | male |
|  | 10.61 (9.97-11.22) | 68.61 (67.55-69.7) | 20.78 (19.54-21.98) | 4.41 (3.64-5.16) | 13.89 (11.48-16.24) | 2021 | female |
|  | 3.83 (3.61-4.05) | 82.8 (82.18-83.41) | 13.37 (12.73-14.03) | 15.89 (13-18.63) | 41.48 (33.84-48.45) | 2021 | male |

All parameters were taken from the latest WHO Global Status Report (cited in the main article).

## Additional File 3: Overall alcohol-attributable fractions for Lithuania 2001-2021, based on different relative risks

### Figure AF1: Overall alcohol-attributable fractions by sex

(a) Females


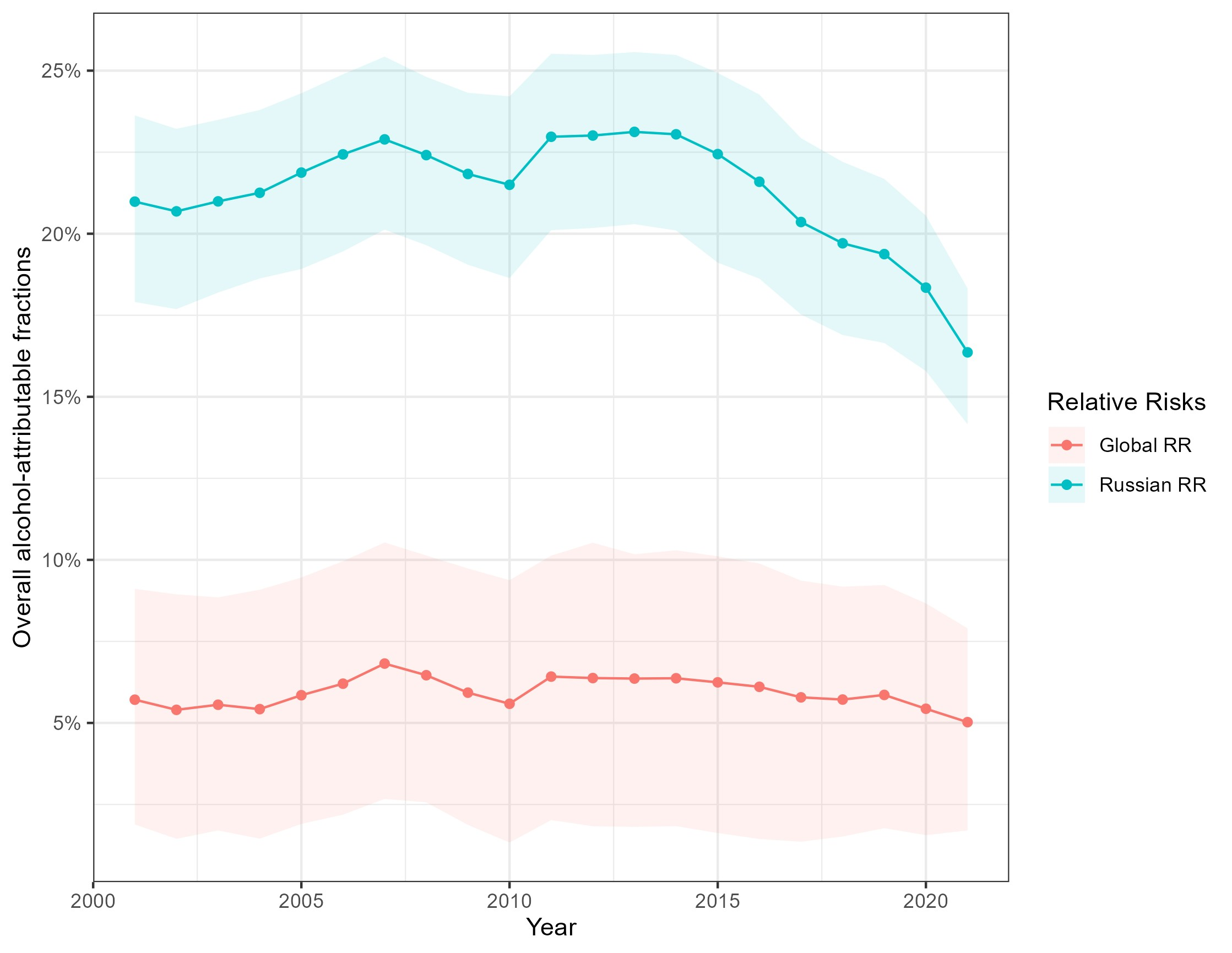


b) Males


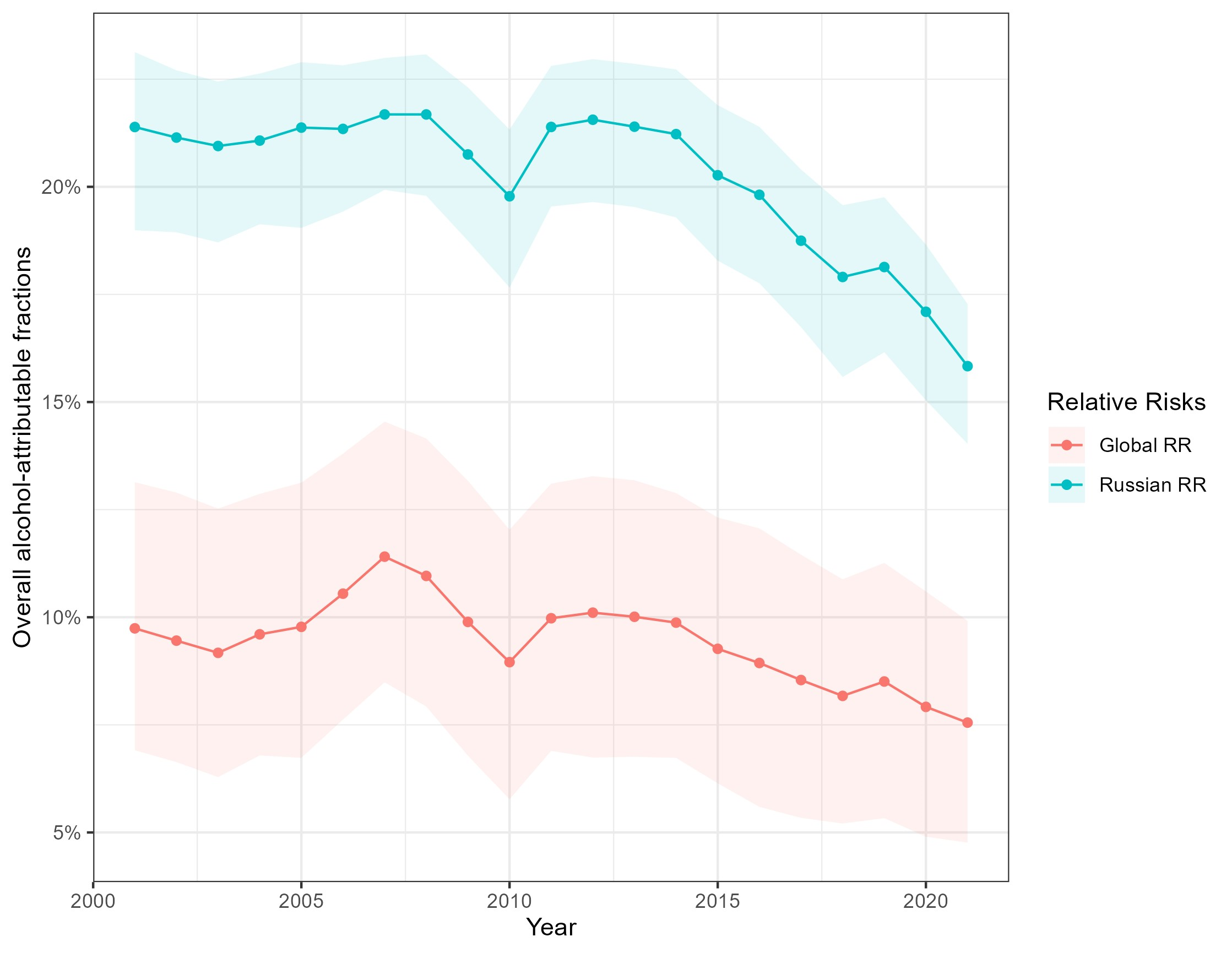


## Additional file 4: Joinpoint analyses

### Joinpoint analysis (Male, Russian relative risk)

| Segment | Joinpoint | 95% CI Lower | 95% CI Higher | Slope  β | Slope  Standard error | Slope  p-value |
| --- | --- | --- | --- | --- | --- | --- |
|  |  |  |  | -0.002 | 0.021 | 0.936 |
| 1 | 2004 | 2003 | 2007 | 0.049 | 0.043 | 0.323 |
| 2 | 2007 | 2006 | 2010 | -0.066 | 0.043 | 0.201 |
| 3 | 2010 | 2009 | 2013 | 0.032 | 0.043 | 0.499 |
| 4 | 2013 | 2012 | 2016 | -0.053 | 0.013 | 0.016 |
| 5 | 2018 | 2015 | 2019 | 0.007 | 0.021 | 0.768 |


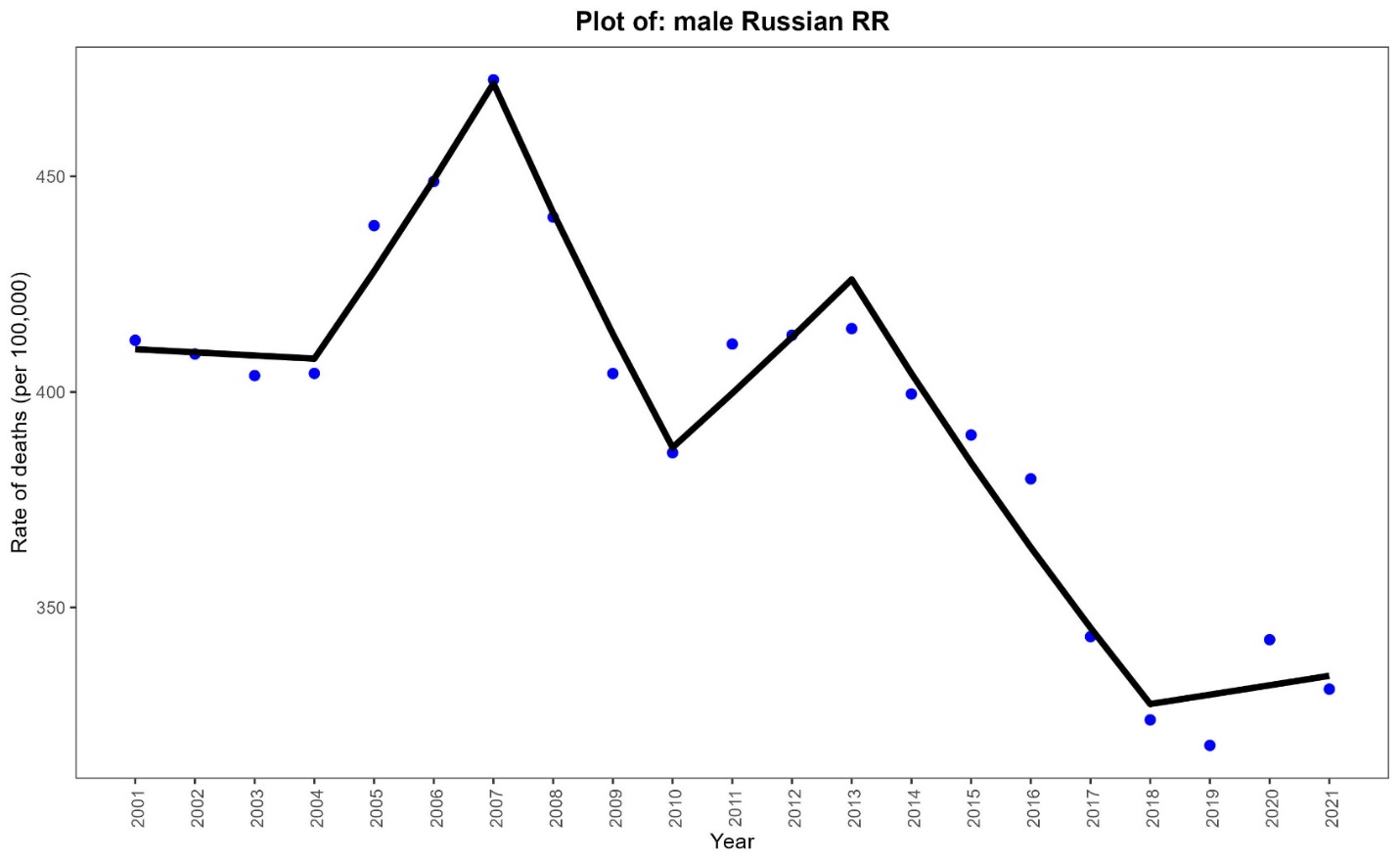


### Joinpoint analysis (Male, Global relative risk)

| Segment | Joinpoint | 95% CI Lower | 95% CI Higher | Slope  β | Slope  Standard error | Slope  p-value |
| --- | --- | --- | --- | --- | --- | --- |
|  |  |  |  | -0.004 | 0.012 | 0.786 |
| 1 | 2004 | 2003 | 2005 | 0.111 | 0.026 | 0.013 |
| 2 | 2007 | 2006 | 2008 | -0.109 | 0.026 | 0.014 |
| 3 | 2010 | 2009 | 2011 | 0.040 | 0.026 | 0.203 |
| 4 | 2013 | 2012 | 2016 | -0.056 | 0.007 | 0.002 |
| 5 | 2018 | 2015 | 2019 | 0.024 | 0.012 | 0.120 |


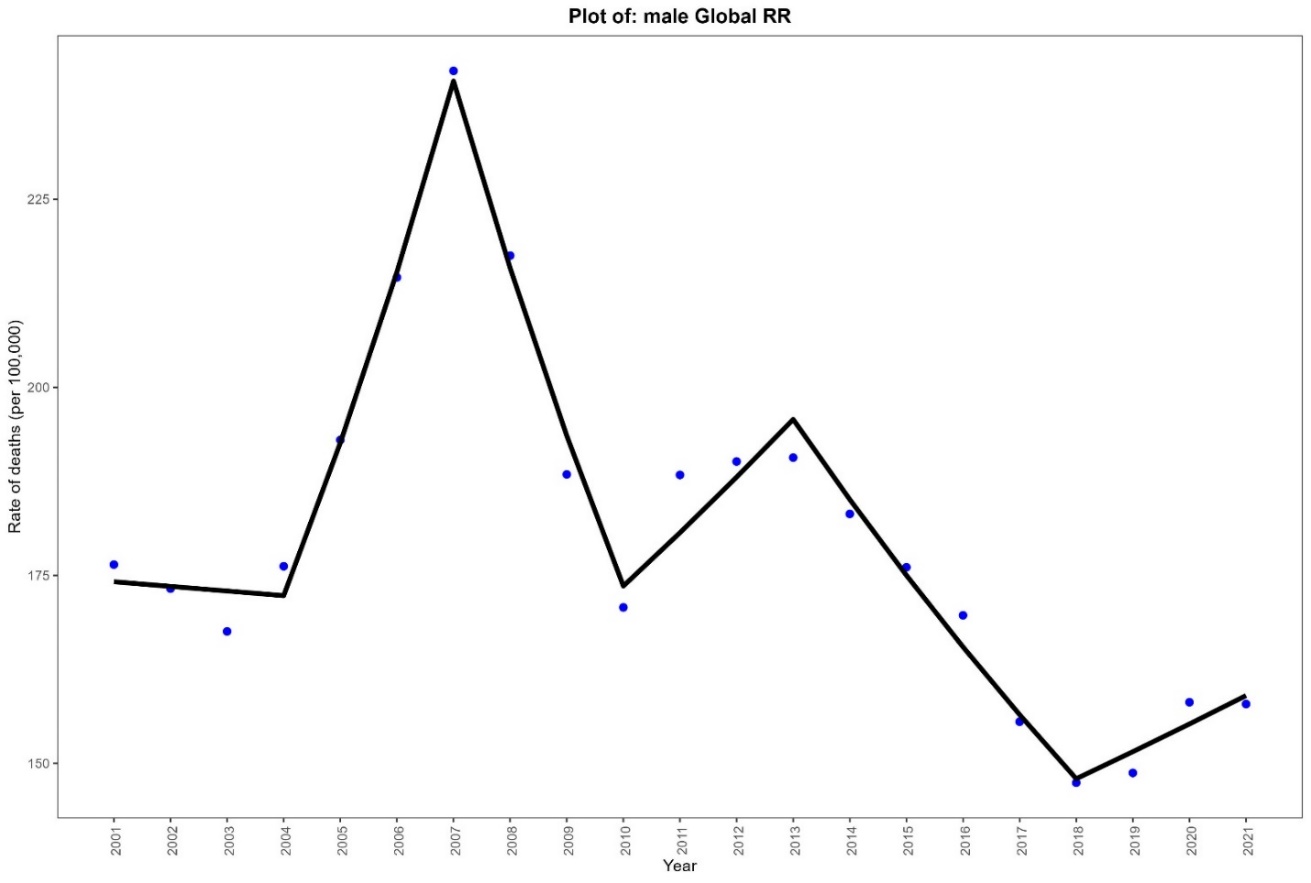


### Joinpoint analysis (Female, Global relative risk)

| Segment | Joinpoint | 95% CI Lower | 95% CI Higher | Slope  β | Slope  standard error | Slope  p-value |
| --- | --- | --- | --- | --- | --- | --- |
|  |  |  |  | -0.017 | 0.017 | 0.379 |
| 1 | 2004 | 2003 | 2005 | 0.090 | 0.035 | 0.063 |
| 2 | 2007 | 2006 | 2008 | -0.064 | 0.035 | 0.146 |
| 3 | 2010 | 2009 | 2011 | 0.053 | 0.035 | 0.207 |
| 4 | 2013 | 2012 | 2016 | -0.024 | 0.008 | 0.035 |
| 5 | 2019 | 2015 | 2019 | 0.037 | 0.035 | 0.350 |


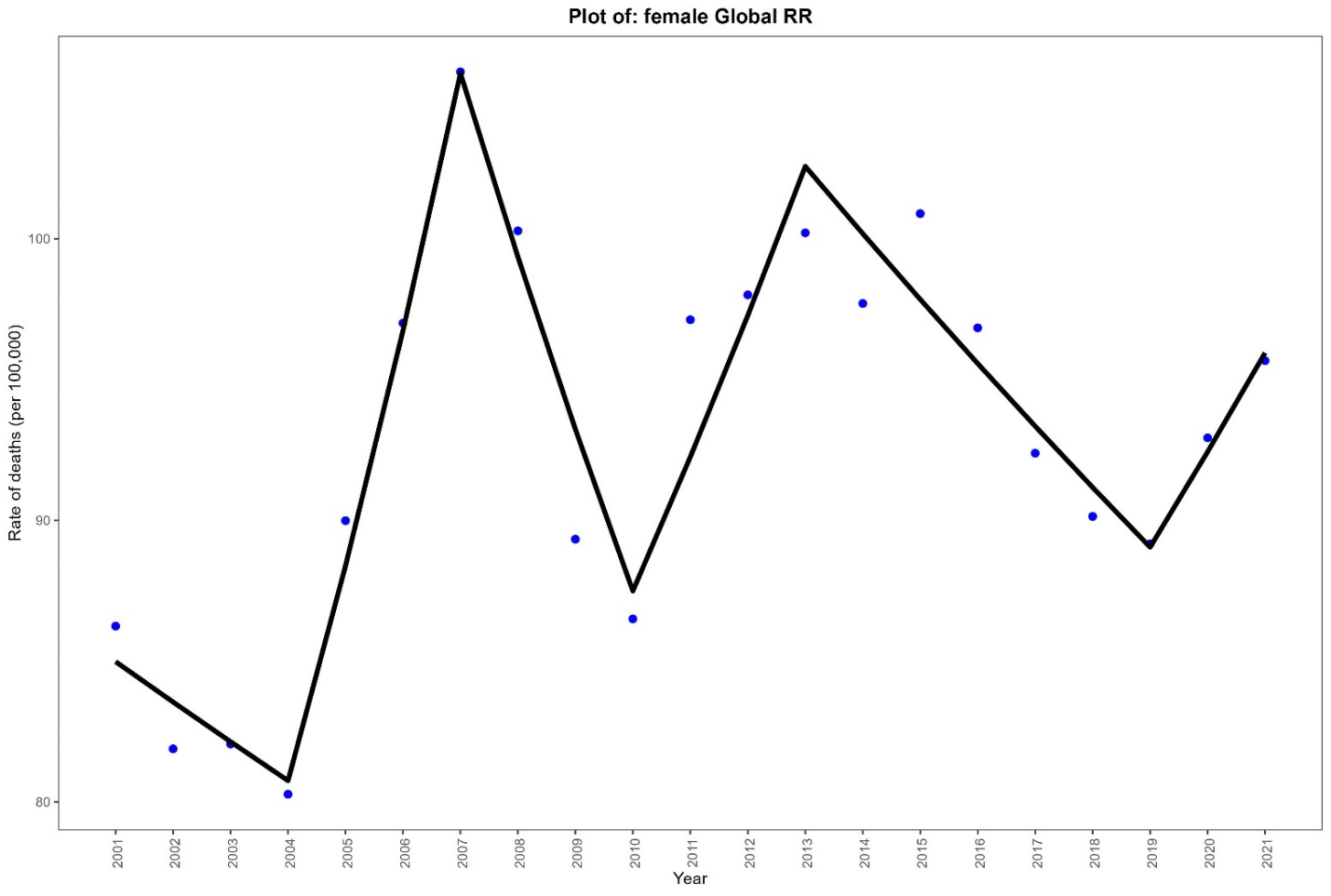


### Joinpoint analysis (Female, Russian relative risk)

| Segment | Joinpoint | 95% CI Lower | 95% CI Higher | Slope  β | Slope  standard error | Slope  p-value |
| --- | --- | --- | --- | --- | --- | --- |
|  |  |  |  | -0.022 | 0.029 | 0.485 |
| 1 | 2003 | 2003 | 2007 | 0.049 | 0.029 | 0.162 |
| 2 | 2006 | 2006 | 2010 | -0.022 | 0.029 | 0.484 |
| 3 | 2009 | 2009 | 2013 | 0.011 | 0.006 | 0.151 |
| 4 | 2015 | 2012 | 2016 | -0.054 | 0.014 | 0.020 |
| 5 | 2019 | 2015 | 2019 | 0.034 | 0.029 | 0.302 |


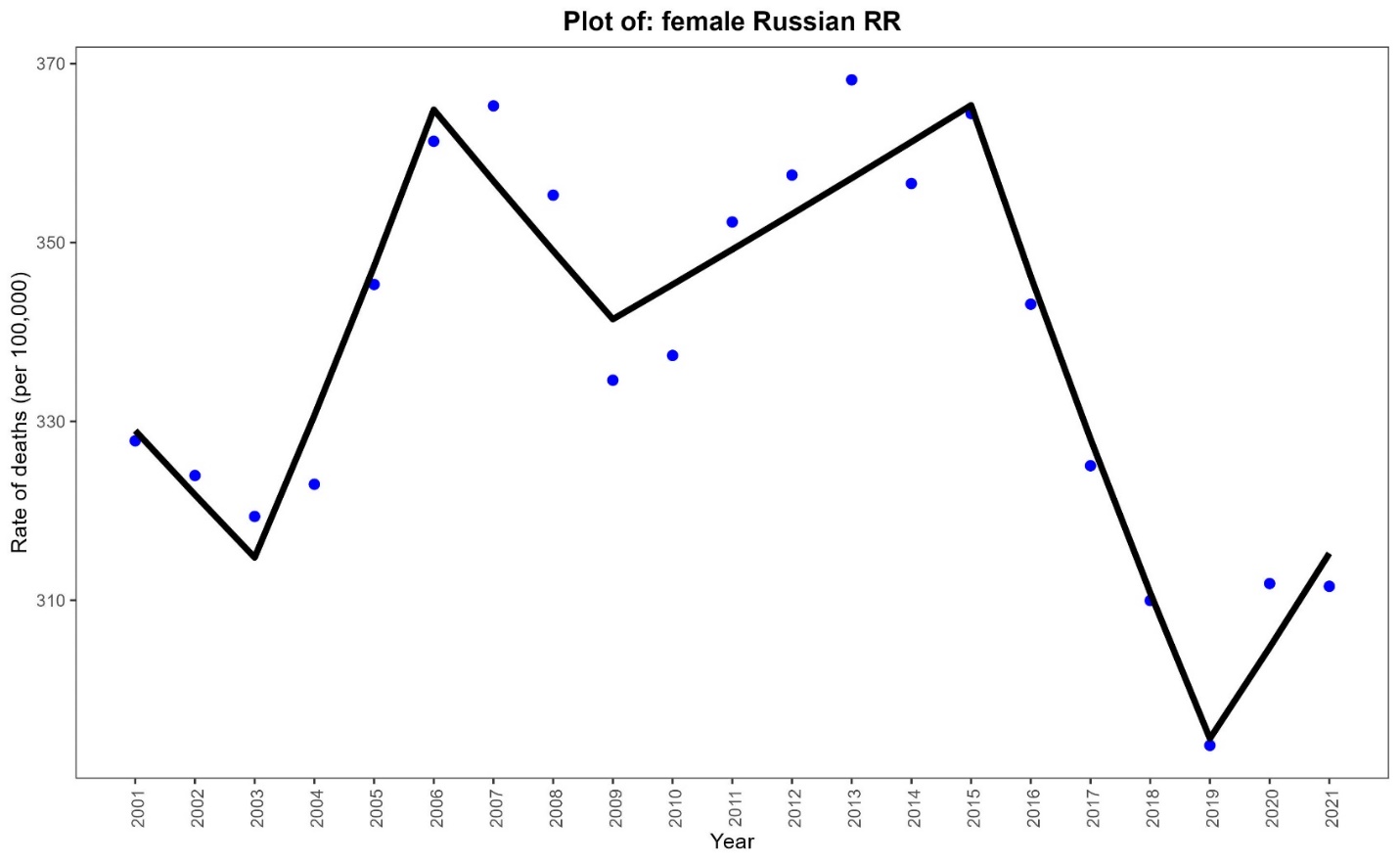


## Additional file 5: Yearly changes in alcohol-attributable mortality, associations with life expectancy

### Yearly change in alcohol-attributable mortality (males, Global RR)

| Predictors | β | Standard error | t-statistic | p-value |
| --- | --- | --- | --- | --- |
| (Intercept) | 4,008.64 | 1,509.38 | 2.66 | 0.0156 |
| year | -1.90 | 0.75 | -2.54 | 0.0201 |

### Yearly change in alcohol-attributable mortality (females, Global RR)

| Predictors | β | Standard error | t-value | p-value |
| --- | --- | --- | --- | --- |
| (Intercept) | 2,144.89 | 1,555.88 | 1.38 | 0.1840 |
| year | -0.90 | 0.77 | -1.16 | 0.2598 |

### Yearly change in alcohol-attributable mortality (males, Russian RR)

| Predictors | β | Standard error | statistic | p-value |
| --- | --- | --- | --- | --- |
| (Intercept) | 10,779.29 | 2,029.06 | 5.31 | 0.0000 |
| year | -5.16 | 1.01 | -5.12 | 0.0001 |

### Yearly change in alcohol-attributable mortality (females, Russian RR)

| Predictors | Β | Standard error | t-value | p-value |
| --- | --- | --- | --- | --- |
| (Intercept) | -791.18 | 477.88 | -1.66 | 0.1142 |
| year | 0.44 | 0.24 | 1.85 | 0.0799 |

### Difference in life expectancy predicted by GDP and difference in alcohol-attributable mortality (males Global RR)

| Predictors | β | Standard error | t-value | p-value |
| --- | --- | --- | --- | --- |
| (Intercept) | 0.35 | 0.34 | 1.02 | 0.3232 |
| GDP per capita | -0.00 | 0.00 | -0.60 | 0.5539 |
| Difference in alcohol-attributable mortality | -0.03 | 0.01 | -4.27 | 0.0005 |
| Durbin-Watson test statistic: 2.09, p = .44 | | | | |

### Difference in life expectancy predicted by GDP and difference in alcohol-attributable mortality (females Global RR)

| Predictors | β | Standard error | t-value | p-value |
| --- | --- | --- | --- | --- |
| (Intercept) | 0.45 | 0.28 | 1.57 | 0.1339 |
| GDP per capita | -0.00 | 0.00 | -1.32 | 0.2034 |
| Difference in alcohol-attributable mortality | -0.05 | 0.02 | -2.63 | 0.0174 |
| Durbin-Watson test statistic: 1.39, p = .041 | | | | |

### Difference in life expectancy predicted by GDP and difference in alcohol-attributable mortality (males, Russian RR)

| Predictors | β | Standard error | t-value | p-value |
| --- | --- | --- | --- | --- |
| (Intercept) | 0.38 | 0.29 | 1.33 | 0.2026 |
| GDP per capita | -0.00 | 0.00 | -1.15 | 0.2673 |
| Difference in alcohol-attributable mortality | -0.03 | 0.01 | -5.66 | 0.0000 |
| Durbin-Watson test statistic: 1.66, p = .13 | | | | |

### Difference in life expectancy predicted by GDP and difference in alcohol-attributable mortality (females, Russian RR)

| Predictors | β | Standard error | t-value | p-value |
| --- | --- | --- | --- | --- |
| (Intercept) | 0.59 | 0.24 | 2.41 | 0.0274 |
| GDP per capita | -0.00 | 0.00 | -2.33 | 0.0324 |
| Difference in alcohol-attributable mortality | -0.03 | 0.01 | -4.07 | 0.0008 |
| Durbin-Watson test statistic: .71, p = .00001 | | | | |

## Additional file 6: Measuring the impact of alcohol control policies by RR type and sex

| Sex | Average percentage changes - global RRs | | |  | Average percentage changes - Russian RRs | | |
| --- | --- | --- | --- | --- | --- | --- | --- |
|  | without alcohol control policies | with alcohol control policies | p-value  (comparing point estimates) |  | without alcohol control policies | with alcohol control policies | p-value  (comparing point estimates) |
| Females | 2.32% | -5.82% | 0.01 |  | 0.93% | -4.61% | <0.01 |
| Males | 1.96% | -9.27% | <0.01 |  | 0.68% | -7.56% | <0.01 |
